# Learning to Pronounce First Words in Three Languages: An Investigation of Caregiver and Infant Behavior Using a Computational Model of an Infant

**DOI:** 10.1371/journal.pone.0110334

**Published:** 2014-10-21

**Authors:** Ian S. Howard, Piers Messum

**Affiliations:** 1 Centre for Robotics and Neural Systems, School of Computing and Mathematics, Plymouth University, Plymouth, United Kingdom; 2 Computational and Biological Learning Lab, Department of Engineering, University of Cambridge, Cambridge, United Kingdom; 3 Pronunciation Science Ltd, London, United Kingdom; Utrecht University, Netherlands

## Abstract

Words are made up of speech sounds. Almost all accounts of child speech development assume that children learn the pronunciation of first language (L1) speech sounds by imitation, most claiming that the child performs some kind of auditory matching to the elements of ambient speech. However, there is evidence to support an alternative account and we investigate the non-imitative child behavior and well-attested caregiver behavior that this account posits using Elija, a computational model of an infant. Through unsupervised active learning, Elija began by discovering motor patterns, which produced sounds. In separate interaction experiments, native speakers of English, French and German then played the role of his caregiver. In their first interactions with Elija, they were allowed to respond to his sounds if they felt this was natural. We analyzed the interactions through phonemic transcriptions of the caregivers' utterances and found that they interpreted his output within the framework of their native languages. Their form of response was almost always a reformulation of Elija's utterance into well-formed sounds of L1. Elija retained those motor patterns to which a caregiver responded and formed associations between his motor pattern and the response it provoked. Thus in a second phase of interaction, he was able to parse input utterances in terms of the caregiver responses he had heard previously, and respond using his associated motor patterns. This capacity enabled the caregivers to teach Elija to pronounce some simple words in their native languages, by his serial imitation of the words' component speech sounds. Overall, our results demonstrate that the natural responses and behaviors of human subjects to infant-like vocalizations can take a computational model from a biologically plausible initial state through to word pronunciation. This provides support for an alternative to current auditory matching hypotheses for how children learn to pronounce.

## Introduction

### Background

A number of learning mechanisms are undoubtedly involved in the development of word and phrase pronunciation, including some forms of imitation. For example, when young children adopt their first ambient word forms they may well recreate them by ‘whole-word’ mimicry [1]. Similarly, ‘progressive phonological idioms’ [2], utterances whose pronunciation is noticeably ahead of or behind a child's general performance, may be recreated as unanalyzed wholes. But it is accepted that at some point the pronunciation of words is learnt by (1) parsing them to identify their constituent speech sounds (which are usually syllable-sized chunks, rather than individual phonemes) and (2) reproducing these elements in their correct order. This form of imitation, the copying of speech sounds in serial order, requires that the infant has already solved the ‘correspondence problem’ [3] for speech sounds. That is, he has developed correspondences between his vocal motor schemes (VMSs) [4], and the speech sounds he hears, such that the result of the former are taken by his listeners to be equivalent (but not necessarily similar) to the latter.

It is generally believed that children solve this correspondence problem by self-supervised auditory matching. In such an account, an infant compares his output of a given speech sound to what he hears produced by others [5], or to what he has heard in the past [6]. He then relies upon his own judgment of their similarity to improve his subsequent performance. In another account, it is supposed that after an infant has discovered sound productions for himself, that these make similar acoustic sequences in the ambient environment especially salient via an ‘articulatory filter’. This makes it easier for him to match and relate some of his productions with those in his linguistic environment [7]. However, these accounts require that the infant is able to compare the acoustic qualities of his own and others' speech sounds. This assumed ability is problematic for a number of reasons [8]. Indeed the apparent lack of acoustic self-regulation of speech output by young infants [9], and even by some adults [10], also speaks against such an acoustic matching mechanism. Furthermore, within the acoustic matching paradigm there is no explanation for the well-known ‘fis/fish’ phenomenon in infant speech, in which a child's speech production (e.g. ”fis”) and the correct L1 form that he hears (“fish”) differ acoustically. The puzzle is that the child's incorrect productions remain stable for longer than would be expected despite the acoustic evidence of a mismatch apparently available to him; a mismatch which he can discriminate in the speech of others and which is often explicitly drawn to his attention by a caregiver [11]–[13].

There have been many previous computational models of speech development; see [14] for a thorough review. These were generally concerned with different issues than those in our work here. In particular they assumed that auditory matching is an unproblematic mechanism for learning to pronounce speech sounds. Some also ignored or downplayed the normalization problem that arises from the different sizes of adult and infant vocal tracts and the inevitable differences in sound qualities that result [15]–[22].

That said, the Asada group have recognized problems with the conventional account and have modeled solutions for vowel learning that use a similar caregiver reinforcement and imitation paradigm as ours [23]–[26]. Overall the main difference between their set of studies and ours is that their focus has been on the initial learning and subsequent development of the infant's vowel qualities, modeling different structural aspects of infant and caregiver interaction. Elija, on the other hand, is a longitudinal model starting from speech sound discovery (both vowels and consonants) and ending with word imitation. We share the same belief that infants are not well equipped to solve the correspondence problem themselves through auditory matching, and that it is within the dynamics of caregiver-infant interaction that a solution can be found.

In this paper we consider an alternative to the mainstream account of auditory matching for how an infant learns to pronounce L1. The alternative account incorporates a main mechanism proposed by Gattegno [27] and elaborated by Messum [8]. We test it through a computational model called Elija [28], and in particular we focus on the role played by caregivers in infant-caregiver interactions. (We note that would have liked to call our infant Eliza, after the female character in Shaw's *Pygmalion* and the musical *My Fair Lady*, who learnt Received Pronunciation from a professor of phonetics. However, Eliza is the name of a famous, pioneering Artificial Intelligence system [29]. Also, we can use pronouns more effectively when we posit a male infant and a female caregiver.)

Elija begins by ‘discovering’ motor patterns of his vocal apparatus that will produce sounds. This is formulated as an unsupervised learning task.

Then Elija interacts with a caregiver, with two effects. Firstly, he retains those motor patterns that generated sound productions that were responded to by the caregiver, and he discards those that were ignored. Thus caregiver response is used as a simple selection mechanism.

Secondly, he solves the bi-directional correspondence problem between the sounds he hears and those that he produces. He does this by making use of the natural, well-attested interaction in which a caregiver responds vocally to an infant's output; an interaction in which imitation is typically involved and understood to be involved by both parties, but undertaken more by the caregiver than the child. Importantly, in this interaction any judgment of sound similarity (or equivalence) that takes place is made by the *caregiver*, and not by Elija. Finally, using Elija's ability to parse input speech utterances in terms of his newly acquired set of equivalents to his own tokens, each caregiver is able to teach Elija to say some simple words by serial imitation in her mother tongue (one of three European languages).

The primary aims of the current study were to demonstrate that Elija could be taught to speak some first words in three languages and to investigate the caregiver behavior that arises during vocal infant-caregiver interaction. Although it is known that in real life infants' babbling (motor pattern discovery) and interaction with caregivers overlap in time, this was not modeled in this version of Elija, which instead ran in three separate stages, for several reasons. These included the need for interaction time with caregivers to be kept within practical limits and the requirement for the same sounds to be heard by all caregivers, so that comparisons could be made across their responses.

### Unsupervised sound discovery by Elija

During speech development, infants progress through several identifiable stages [30]. Within a few months of birth, they are producing quasi-vowels and cooing. Over the next few months they start marginal babbling; producing vowels, raspberries and squeals. Canonical babbling can start from 5 months. This initial development appears to arise from an infant's unsupervised experimentation with his speech apparatus.

**Figure 1:**
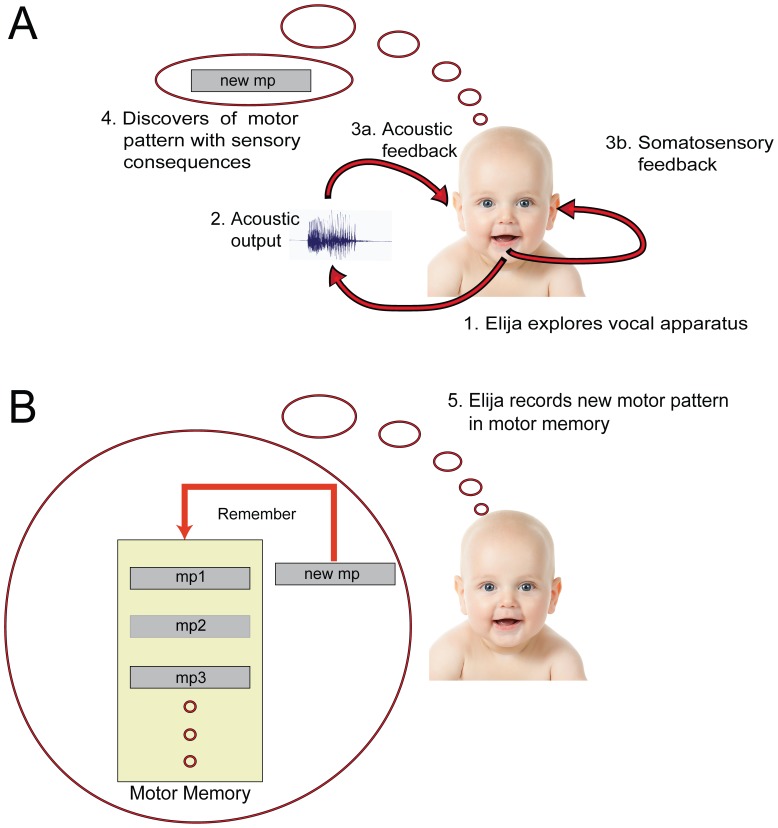
Elija learns from babbling. Panel A: Elija's (virtual) motor activity moves his vocal apparatus and he can explore the sensory consequences of this activity (1). This will sometimes result in the generation of acoustic output (2). The presence of acoustic output can be noticed by Elija (3a), as can other somato-sensory consequences of the vocal tract movement, such as touch arising from vocal tract closure (3b). The exploration can lead to the discovery of a motor pattern (4). Panel B: A discovered motor pattern is stored in motor memory (5).

To model this natural development, Elija starts by exploring his vocal apparatus. He creates motor activity that repositions his vocal articulators from their resting state and he evaluates the sensory consequences [31]: sometimes this results in the generation of acoustic output and sometimes somato-sensory effects such as touch arising from vocal tract closure. Acting on this feedback, he tries to improve his motor actions in accordance with a reward scheme involving multiple terms chosen to be developmentally plausible. In this way, his exploration leads to the development of motor patterns for the production of sounds that may later turn out to be useful as speech sounds. (NB: In real infants, motor patterns that produce sounds and have stabilized are described as vocal motor schemes (VMSs) [4].) The motor pattern discovery process used in Elija is illustrated in Fig. 1.

### Elija makes use of caregiver responses

Exposure to a language is necessary for a child's development of pronunciation, and it is clear that there is always interaction with learned speakers during L1 word adoption. In our account, interaction is necessary before this, in the development of a capacity to perform word imitation. (We note that in real life the processes that support speech development overlap. Many things happen in parallel. For clarity of exposition, here we are describing events as if they occur in sequence). The process starts as an infant's sound production begins to attract his caregiver's attention. His development at this point relies on a caregiver's willingness to vocally ‘imitate’ him, as observed naturally [32], [33]. During these interactions, both parties understand that she is imitating him [33], [34], so he is aware that his caregiver must regard his and her utterances as equivalent in some way.

Although not explicitly instructed to do so, in our earlier experiments we found that a single (male) experimental caregiver found it natural to respond to those of Elija's utterances that he judged to be similar to sounds that he could easily produce himself [28]. In the great majority of cases he reformulated Elija's utterances into well-formed L1 speech sounds. Here we further examine this observation with eight speakers of three languages.

The caregiver's responses affect Elija in two ways. Firstly, a response reinforces the production of the motor pattern that provoked it, whereas its absence discourages further use of this motor pattern. Secondly, Elija is allowed to associate his motor patterns to his caregiver's responses. We argue that both effects reflect the likely reality of speech development. The first was reported, for example, by Pelaez et al. [35]. The second is reasonable, given that the presentation of a response immediately after an infant's vocal action provides a favorable condition for associative learning [36]. Such a response provides a real child with an interpretation of his production; given the imitative context in which it occurs, he is informed that, in his caregiver's judgment, the output from his motor pattern and her response are equivalent in some sense. Importantly, this does not require an infant (or Elija) to make a judgment of similarity between his and her output. Therefore, at this stage of his development no sophisticated perceptual expertise is required on an infant's (or Elija's) part. (Such expertise, needed for solving the normalization problem, has to be assumed by conventional imitative theories).

**Figure 2:**
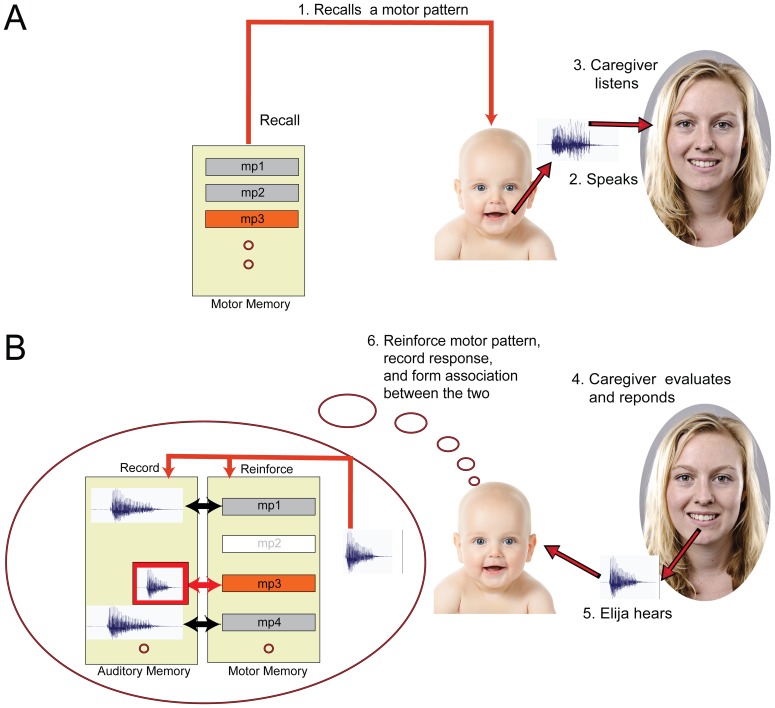
Tutored equivalence. Elija learns to pronounce using caregiver responses, which reinforce some utterances and allow him to associate his motor patterns to adult L1 speech output. Panel A: Elija first recalls a motor pattern, e.g. motor pattern 3, (1) and uses it to make an utterance (2). The caregiver hears the sounds (3). Panel B: The caregiver may reformulate it using her L1 interpretation of Elija's sound production (4). Elija hears the caregiver's response (5). Aware that he is being imitated, Elija takes the caregiver's utterance as equivalent to the output from his motor pattern, which reinforces motor pattern 3 and associates it with the response (6). If a motor pattern is not responded to, it will be deselected and have no link to an auditory memory (e.g. motor pattern 2).

Fig. 2 shows how this tutored equivalence paradigm operates. Elija first recalls a motor pattern that he previously discovered by exploration. He then uses it to drive his vocal apparatus and generate an utterance in the presence of his caregiver. The caregiver hears the sounds and if she feels it is natural to respond, she is free to do so. During this period, Elija is attending to the caregiver, hears any response she makes and associates them. If a motor pattern is not responded to, it will be deselected and no link to an auditory memory is created.

### Serial imitation of speech sounds

After Elija has associated some of his motor patterns to his caregiver's responses (which, as we will show, are generally reformulations of his output into L1), he has the information needed to parse strings of input sounds in terms of sounds he has heard before and to respond using his associated motor patterns. Thus after the first interaction stage, a caregiver is able to teach Elija to pronounce words by his serial imitation of their component speech sounds. Of course, Elija's ability to perform well at word imitation relies on the extent to which his repertoire of motor pattern/reformulation correspondences covers the sounds that make up the words his caregiver is trying to teach him, and on the quality of his motor pattern outputs within these pairings.

**Figure 3:**
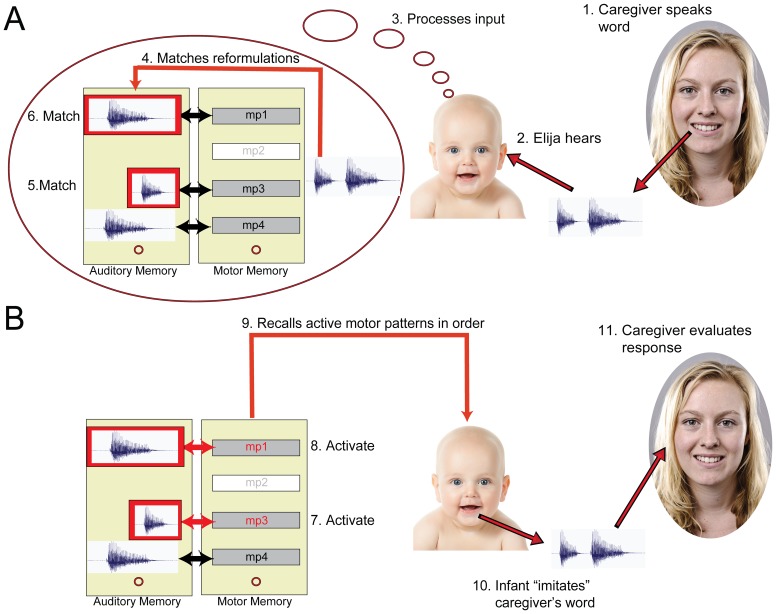
Learning to pronounce a word using serial imitation of its component speech sounds. Panel A: The caregiver says a word, in this case consisting of two distinct speech sounds (1). Elija hears the caregiver's utterance (2) and starts to process it (3). This involves performing an auditory matching to previously heard responses (4). Matching auditory memories are then activated in sequence (5,6). Panel B: The activated auditory memories in turn activate motor pattern 3 and motor pattern 1 in motor memory (7,8). They are then recalled in sequence (9) resulting in the generation of output speech (10), which constitutes Elija's imitation of the caregiver's utterance. Finally the caregiver hears and can evaluate Elija's response (11).

Fig. 3 gives an overview of how this mechanism is implemented in the Elija model. First, the caregiver speaks a word that she has chosen to teach Elija. He hears the caregiver's utterance and segments it into syllable-size constituent speech sounds. He then performs an auditory matching between these incoming sounds and all the caregiver responses he previously associated to his motor patterns. When matches to auditory memories are found, the associated motor patterns in motor memory are activated. These motor patterns are recalled in sequence and used to drive his vocal apparatus, resulting in the generation of output speech. This constitutes his imitation of the caregiver's word, and can be heard by the caregiver.

**Figure 4:**
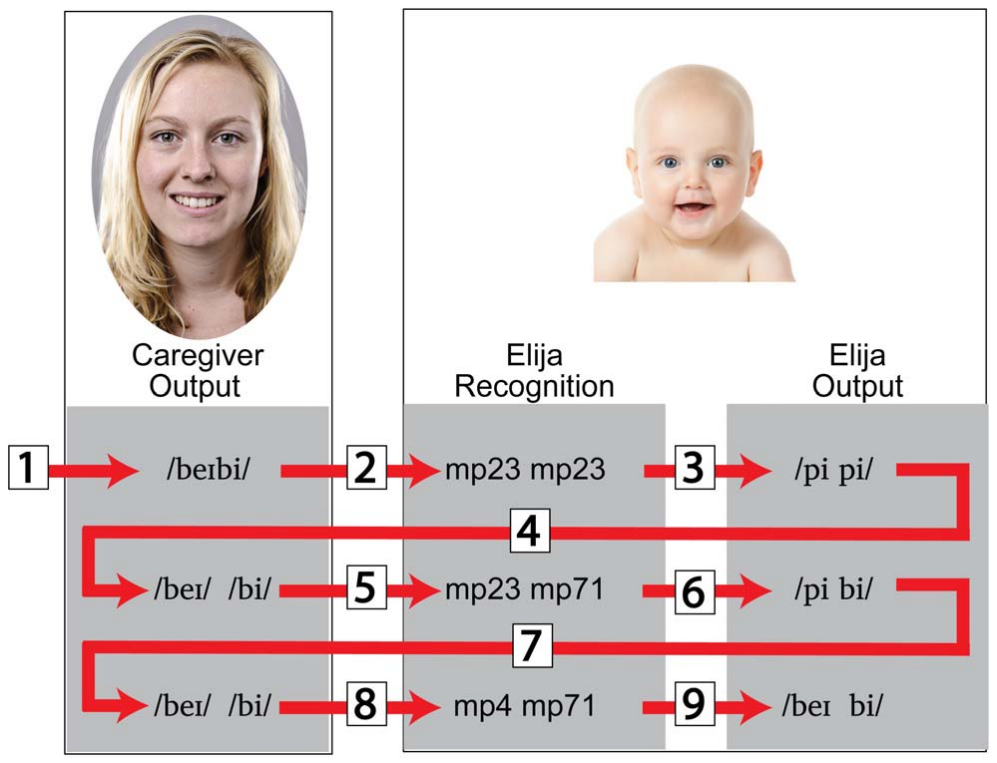
Repetitive interaction loops in word learning. The caregiver first says a word (1). Elija recognizes its component sounds in terms of sounds he has heard before (2). Using the associated motor patterns, he then generates speech output (3). The caregiver evaluates Elija's response and, if not satisfied, may say the word again, perhaps more clearly (4). Elija performs recognition again (5) and generates a different response (6). This process can continue (7–9), until (as in this case) the caregiver decides that performance is satisfactory. Alternatively, if the task is not productive, the caregiver can give up and try to teach Elija a new word.

However, this isn't necessarily the end of the process. Elija and his caregiver are allowed to engage in repetitive loops, as shown in Fig. 4. When the caregiver hears Elija's response, she may not be satisfied with his attempt. She can then say the word again, perhaps more clearly and in a way she thinks Elija can more easily understand. This gives Elija another opportunity to learn the word, which he again does by trying to recognize her sounds and generating a response. This procedure continues until the caregiver either decides that performance is satisfactory or, if his attempts are not successful, gives up and tries to teach Elija a different word.

## Materials and Methods

We model an infant as a computational agent, Elija, who has no *a priori* articulatory or perceptual knowledge of speech [28]. More details of his operation are provided in the extended methods section in Appendix S1 in File S1.

**Figure 5:**
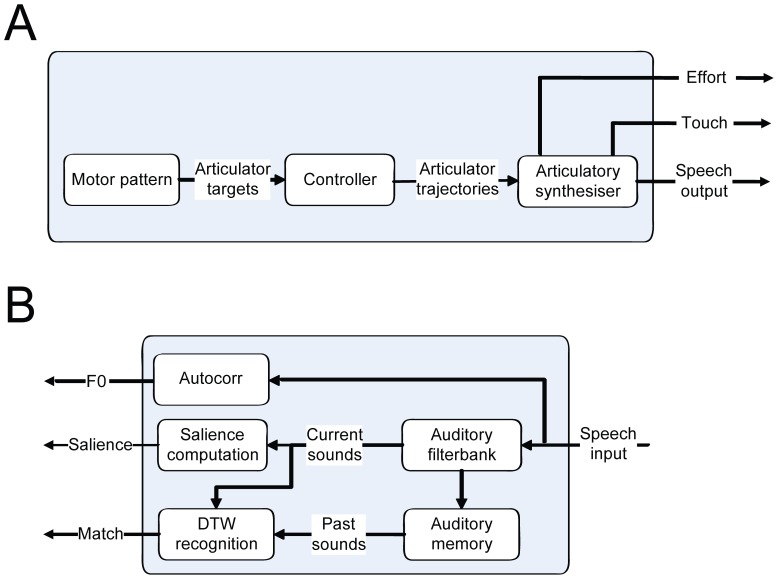
Elija's motor and perceptual systems. Panel A: Elija's motor control system incorporates a Maeda articulatory speech synthesizer. A motor pattern is a sequence of articulatory targets for the synthesizer's control parameters. These are interpolated by a controller, which assumes that the articulator movements follow 2^nd^ order critically damped trajectories. The resulting sequences of time-varying parameter vectors drive the synthesizer. This potentially generates acoustic output, which is played out via a loudspeaker. In addition, the effort in the production is estimated and any closure of the vocal tract is reported. Panel B: Elija's perceptive system. A USB microphone first digitizes the acoustic input. Autocorrelation analysis is applied directly to the waveform to estimate its fundamental frequency F0. An auditory filter bank provides pre-processing of the input. Further processing estimates signal salience, which is used by the reward mechanism. Pre-processed input can be recorded in auditory memory and also compared against past memories using a speech sound recognizer that is based on DTW.

The main features of Elija's motor system are shown in Fig. 5A. Elija has a speech production capability based on a modified Maeda articulatory synthesizer [37], [38]. This is driven by a motor system in which representations of motor actions are akin to the gestural score used in the Task Dynamics model [39]. A motor pattern is a sequence of articulatory targets for the synthesizer's control parameters. A controller assumes that the articulator movements follow 2^nd^ order critically damped trajectories and interpolates between these targets. The resulting sequences of time-varying parameter vectors drive the synthesizer. This can lead to acoustic output played out via a loudspeaker.

A schematic of Elija's perceptive system is shown in Fig. 5B. Elija's hearing system receives input from a Rode Podcaster USB microphone. Autocorrelation analysis is applied directly to the input waveform to estimate the fundamental frequency F0. An auditory filter bank provides initial pre-processing of the input [40]. Our implementation is based on the gammatone-like spectrograms implemented by Ellis [41].

Analysis of Elija's own acoustic output is carried out directly on the digitized signal from the synthesizer, although in principle this could also be achieved by passing acoustic output back from the loudspeaker via the microphone. Further processing estimates signal salience, which is used as a component in Elija's reward mechanism. Pre-processed input can be recorded in auditory memory and also compared against past memories using a speech sound recognizer that is based on Dynamic Time Warping (DTW) [42]. This enables Elija to discriminate different speech sounds.

### Maeda articulatory synthesizer

In our implementation of the Maeda articulatory synthesizer [37], [38], ten parameters are used to control the vocal apparatus, the first seven being articulatory: P1 Jaw position, P2 Tongue dorsum position, P3 Tongue dorsum shape, P4 Tongue apex position, P5 Lip height (aperture), P6 Lip protrusion, P7 Larynx height. In addition, an LF voice source model was added to give control over a voiced excitation model [43]. (LF, named after the authors Liljencrants and Fant, is a four-parameter model of glottal flow.) This makes use of two additional parameters: P8 Glottal area, and P9 Fundamental frequency. In the original VTCALCS implementation a velo-pharyngeal port was added to the basic model and its opening is controlled using parameter P10 Nasality. Thus the Maeda synthesizer enabled Elija to produce both oral and nasal sounds. After the vocal tract profile is specified by the elementary articulator parameters, an equivalent digital filter is computed and used to filter the excitation from the voice source and other noise sources. Fricatives are simulated in the model by injecting noise at locations in the vocal tract where turbulent airflow is predicted.

In our experiments, the synthesizer operated with an output-sampling rate of 24 kHz. To approximate an infant vocal tract adequately for the purposes of these experiments, the model's default physical dimensions, which originally reflected the sizing of an adult female vocal tract, were scaled down by a factor of 0.8.

Similarly, the mid-range of the fundamental frequency was shifted from 210 Hz to 400 Hz. We added proprioceptive feedback of lip and tongue contact, which was generated at times when the vocal tract tube cross-sectional area reached zero. Elija was implemented in C++ and all other analyses were written in Matlab (Mathworks Inc, Natick MA, USA) running on a PC. Acoustic output was played to the caregiver from the PC's inboard DAC output via a pair of active loudspeakers.

### Modeling motor patterns and articulator dynamics

As in a previous implementation of Elija [28], motor actions were modeled in a way akin to the gestural score used in the Task Dynamics model [39] and movement of Elija's articulators between targets was implemented by assuming 2^nd^ order dynamics that follow critically damped trajectories [15]. In this work we extend our former approach and the dynamic properties of different vocal tract articulators are now no longer all grouped together. Rather they are given individual properties (see below). We note that other approximations to articulator movements could also be made, e.g. using a minimum jerk trajectory, which is often used to describe human arm movements [44].

In Elija, a motor pattern can be a sequence of up to three different sub-patterns. Each sub-pattern specifies parameters needed to control the vocal apparatus and contains a 10-element target vector, a 10-element starting time vector and a 10-element duration time vector specifying the how long a target is maintained. There is also a single overall transition speed scaling parameter 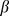. Thus each sub-pattern consists of 31 elements.

Each component target vector gives rise to movement of the articulators from their current state towards their new target values. As stated above, such articulator movement follows a critically damped trajectory, leading to articulator movement towards its target without overshoot [15]. We compute the trajectory of each control parameter using the equation:
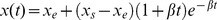
Where 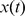 is the parameter value at time 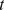, 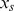 is the starting point, 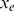 is the end point (target value), the constant 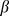 is given by the relation 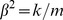, where 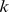 is the spring constant and 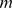 is the associated mass of the dynamical system.

The value of 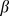 associated with the different vocal tract articulator parameters is matched to their dynamic properties. For movements of the articulators during vocalic, sonorant and fricative sound generation, a value of 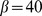 is used, since it matches typical human articulation speeds well. However, during plosive sound generation transitions are much faster due to the rapid release of air pressure at the point of vocal tract closure. To account for this phenomenon, transitions following closure have their associated 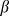 value increased to 160. This leads to the generation of more realistic plosive sounds.

### Unsupervised sound discovery

Elija's discovery of sound-generating motor patterns under developmentally plausible influences is formulated as an optimization problem that operates without caregiver involvement, and is an extension of previous work [31]. The modeling of autonomous exploration has recently become an area of interest for several researchers, including those working in the field of developmental robotics [45]–[50]. We note that Elija uses both intrinsic and extrinsic reinforcement, as described by Warlaumont [51], during his sound discovery and refinement process.

As before, our objective function for the optimization of motor patterns includes terms that encourage salience and diversity and discourage motor effort. In addition, we now include a term that discourages the discovery of ‘sensitive’ motor patterns, as explained below. The continuous scalar reward value 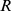 computed in the objective function of the algorithm is given by:
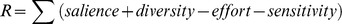
The salience term encourages Elija to find motor patterns that generate sensory consequences. Sensory salience was estimated by combining several components: averaged weighted low and weighted high frequency power over the duration of the motor pattern and the average touch signal.

We assume that a human infant can and does selectively focus his attention on these different aspects of sensory feedback. Elija does so by changing the relative contribution of the components of salience. Attending to acoustic power at lower frequencies will favor the discovery of configurations that lead to vowel production, while attending to acoustic output with a dominant high frequency component will favor the discovery of fricatives. Attending to touch will favor configurations used in consonants, such as where the lips are closed or the tongue makes contact with the teeth or the roof of the mouth.

The diversity term is included in the objective function to encourage the discovery of a range of motor patterns that lead to different sensory consequences. That is, it encourages the discovery of novel patterns that are different from the previous ones found. Diversity was computed as the weighted sum of three components in acoustic, tactile and motor pattern space. In each of these spaces, the minimum distance arising from the current motor pattern to all previous motor patterns was calculated. The weighting affected the class of motor patterns discovered. A strong tactile weighting biased the optimization to the discovery of distinct plosive articulations, whereas a strong acoustic weighting biased the optimization to the discovery of acoustically distinct vocalic and fricative sounds. We note that such explicit weighting is not strictly necessary, since the diversity term will by its very nature result in active exploration. However its inclusion does speed up the computational process.

The effort required to execute the motor pattern makes a negative contribution to the objective function. Effort was determined by a combination of the cost of movement and the loudness of the voiced excitation. The cost of movement was calculated as the weighted sum of articulator speeds over the duration of the motor pattern. Loudness of the voiced excitation was estimated by summing the voicing contribution to Maeda parameter P8 over the duration of the motor pattern. The effort term is important because if no penalty is included for voicing loudness, the optimization generally finds a solution with the voicing parameter set to maximum, because this always maximizes sensory salience. We note that the effort term could be enhanced, for example by incorporating ‘toil’ (relating to the deformation of the vocal tract) as defined by Yoshikawa et al [24].

A sensitivity term is included in the objective function to penalize the discovery of motor patterns that create sounds that can only be generated by very accurate articulations. More specifically, motor pattern sensitivity relates to how much the acoustic output of a given articulation changes when the motor pattern is subject to local perturbations:
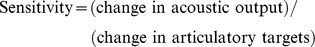
Sensitivity issues affect the discovery of vowels. Given that some variability is found in speech production and is a feature of the learning process, insensitive articulations will more reliably lead to an acceptable intended acoustic output than sensitive ones. There is reason to believe that very sensitive articulator configurations are not utilized in speech production, as addressed in Steven's Quantal Theory [52] and Gunnilstam's Theory of Local Linearity [53]. Both hypothesize that preferred regions of articulation in speech production exist and that there are, for example, regions of articulator space that provide a natural location for vowel sounds. The sensitivity of the acoustic realization of a given motor pattern was computed by first individually positively perturbing the parameters P1 to P5. A perturbation corresponding to 5% of the full parameter range was used (i.e., a value of 0.1 was added to each Maeda parameter). All other parameters were set to constant values across all motor pattern vectors to avoid added variability in acoustic output. The output time waveforms for the unperturbed motor pattern and for each of the 5 perturbed motor patterns were generated using the Maeda synthesizer and were then analyzed using the auditory filter bank. The distance between the auditory representation of each perturbed motor pattern and that of the unperturbed pattern was computed. The overall sensitivity for the given motor pattern was then taken as the square root of the sum of squares of the 5 components. The perturbed patterns were only used to assess the sensitivity of the pattern under investigation and were not stored in memory.

### Running motor pattern discovery

In the Elija model, motor pattern discovery starts by setting the elements of the motor pattern to random values drawn from a uniform distribution over their valid range (−1 to 1). Motor pattern solutions are then found using 3 iterations of a Quasi-Newton gradient descent algorithm, as implemented by the Matlab function fmincon (which finds a constrained minimum).

Since this study investigated sound and subsequent word learning, several steps were employed to ensure that Elija discovered a wide range of suitable motor patterns within a reasonable time. Using single target motor patterns, separate optimization runs were employed with an emphasis on low frequency power (for vowels), high frequency power (for fricatives) and touch (for plosives). To increase the variety of sounds, voicing was explicitly enabled or disabled in each plosive and fricative articulation (that is, this operation was not carried out automatically by the optimization procedure). Similarly, closures were generated with or without opening of the velo-pharyngeal port, creating nasals or plosives respectively. We note that during motor pattern discovery active learning was always present. Therefore, although the *a priori* biasing was used to reduce exploration times, if the motor pattern discovery process had been allowed to run for long enough it would have found a comparable final set of consonants and vowels autonomously, without making such interventions, as was achieved in our previous study [28].

To limit the overall number of motor patterns, clustering was used to reduce the occurrence of articulations that were similar. Such clustering maintained variety, but limited redundancy and ensured that there was no subsequent combinatorial explosion of C and V configurations when sequences were generated (see below). The clustering of plosive configurations was performed directly on motor patterns using a standard K-means algorithm. Vocalic and fricative sounds were clustered acoustically using a modified version of the same algorithm, using dynamic time warping (DTW) as its metric of similarity [28]. The total number of motor pattern clusters and categories were set by hand to limit their number. Again we note that clustering would be unnecessary if long interaction times with caregivers were acceptable. Ideally, all the raw motor patterns discovered by the optimization search would have been used and evaluated by the caregiver, but this would have required much longer periods of interaction.

The number of vocalic sounds discovered was limited to 15, the number of plosives was limited to 15 and the number of fricatives limited to 10. As a result, the subsequent interaction experiments could be carried out within 2–3 hours per caregiver.

### Expanding motor pattern variety

By concatenating the simple motor patterns discovered by the optimization procedure, Elija can generate more complex utterances that are potential speech sounds. Single articulations were combined to generate VVs (sounding similar to true diphthongs), CVs, CVVs and VCs. More specifically, Elija generated CV (C_v_V, C_u_V, F_v_V, F_u_V, NV), VC (VC_v_, VC_u_, VF_v_, VF_u_, VN) and VV tokens, where N  =  voiced nasal consonant, C_v_  =  voiced consonant, C_u_  =  unvoiced consonant, F_v_  =  voiced fricative, F_u_  =  unvoiced fricative. Longer sequences were in principle possible, but not used in the current study. Again we note that the combination of simple motor patterns into complex motor patterns was only performed to reduce the time needed to discover motor patterns. If the motor pattern discovery process had been allowed to run longer and to find multiple target motor patterns, the complex motor pattern discovery process could operate fully autonomously as in our previous study [28].

After the authors removed implausible sounds by hand (for example, synthesizer artifacts such as clicks), Elija had discovered 927 motor patterns, which could be used for the first response experiments.

### Ethics statement

After providing written informed consent, a total of 8 subjects (3 male, 5 female) played the role of Elija's caregiver in separate experiments. All subjects were native adult speakers of the languages in which they interacted with Elija. We note that no children were involved in this study. The Cambridge Psychology Research Ethics Committee at the University of Cambridge approved the experimental protocol.

### Experiments

The first experiment investigated caregiver responses in three different languages using all 8 subjects. We examined variability of responses within the speakers of the same language. The second experiment investigated the variability of the responses from a single English speaker over 4 sessions. The third experiment investigated word learning by Elija through serial imitation and made use of 6 of the subjects (2 in each language), each of whom had previously responded to Elija's output in Experiment 1.

### Experiments 1 & 2: First caregiver interactions with Elija

The first experiments investigated caregiver responses to Elija's 927 motor patterns. The caregivers were instructed to close their eyes and to imagine that they were interacting with a human infant. They were not given any information about the child's age, or shown a picture of an infant. They were asked to either respond or not respond ‘naturally’ to what they heard.

**Figure 6:**
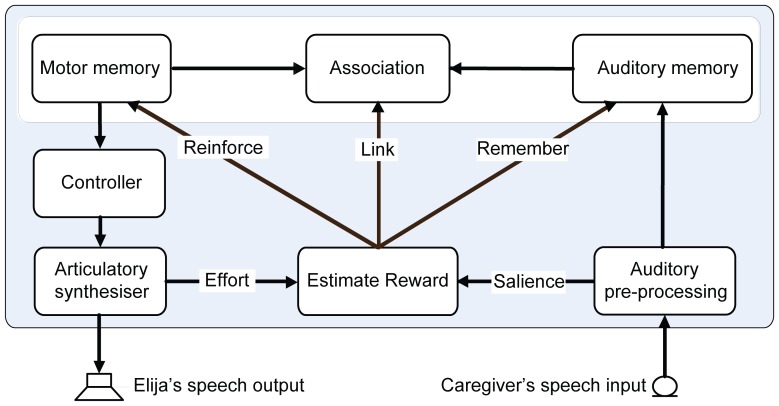
Formation of associations between motor and auditory memories. Elija generates an acoustic output by using a previously discovered motor pattern. After production, Elija records any potential response from the caregiver. If the caregiver responds, the auditory salience of this response will contribute to a reward signal. This will cause Elija to remember the speech input response, reinforce the motor pattern and also build an association between the two.

The caregivers prompted Elija to generate an utterance by pressing a key on the keyboard. Elija then executed a motor pattern, which generated a sound to which his caregiver might respond. Elija listened for 3 seconds after each of his productions and recorded any vocal response the caregiver chose to make. Elija detected if the caregiver responded using a simple speech detection mechanism. This involved determining if the short-term power in any acoustic response exceeded background noise level. When a response was detected, the motor pattern responsible was retained and an association between the response and the motor pattern was created (Fig. 2). When a caregiver ignored a sound, the underlying motor pattern disappeared from Elija's motor pattern repertoire. Fig. 6 shows how this process forms associations between motor and auditory memories: immediately after executing a motor pattern, Elija captures any response from the caregiver in auditory memory, retains the motor pattern in motor memory and builds an association between the two.

We note that Elija did not change his motor patterns as a result of interaction with his caregivers (the same approach as taken by Miura et al. [25]). They were only optimized during the initial self-supervised learning stage. This study compared the behavior of different caregivers and it was therefore important that all caregivers heard the same sounds so that comparisons of their responses could be made.

### Experiment 3: Word learning mechanisms in Elija

After Elija had learned the associations between his productions and adult forms made in response, he could attempt to imitate novel utterances made by the caregiver (Fig. 3). He parsed them in terms of previously heard responses and since these sounds had associations with his motor patterns, this process provided him with candidates for the reproduction of words by serial matching of their component sounds.

To implement the recognition mechanism, Elija employed a template-based dynamic time warping (DTW) recognizer [54], running with an auditory gammatone filter bank front-end [40]. Such DTW recognizers typically operate by matching spectral representations of input speech with another set of such representations that correspond to the vocabulary of the recognizer. The latter are simply ‘templates’ or good examples of the sounds in its vocabulary. The template that gives the closest match is then taken as being the classification of the input sound. In the Elija model, the DTW recognizer used the caregiver's responses as its sound templates. However, since words could contain several basic speech sounds concatenated together, a segmentation mechanism was used to present them individually to the template-based recognizer. This required that the caregiver spoke with pauses between syllables. Segmentation into separate utterances was achieved by finding regions in which the short-term power of the signal exceeded the background noise level.

In practice, a two pass recognition scheme was used to ensure real-time operation [28]. In the first pass, the recognizer operated by using 100 templates selected as the cluster centers of all responses. In the second pass, all the members of the best 5 clusters were used as templates. We note here that because Elija only matched caregiver speech with caregiver speech, there was no normalization problem for the classifier to solve.

During this experiment, Elija played out the motor patterns he had identified by the recognition process. Elija was given the ability to produce an intonation contour on each word resembling that of the caregiver, which made his attempts at word imitation sound more natural. To achieve this, the fundamental frequency contour for each separate speech sound was computed and approximated to a straight line using linear regression. The start and end frequencies were extracted and then mapped onto the range of the Maeda synthesizer voice source F0 parameter by assuming a linear scaling between the (−0.9, 0.9) parameter range and a frequency range of either 100 Hz to 300 Hz or 150 to 400 Hz, for a male or female caregiver respectively. The duration of the speech sounds in the caregiver's speech was estimated and the values were limited to fall within the range of 250 ms to 600 ms. The F0 and duration parameter values were then used to set the fundamental frequency and duration parameters in the appropriate motor patterns. All interactions, including Elija's internal recognition process, were recorded to document the development of his pronunciation.

The word-learning task was run on a PC and a graphical user interface provided the caregiver with a word from a list, generated from words typically spoken by young children in the caregiver's language. The caregiver first pressed the ‘Go’ button and spoke the word. Elija then repeated it using his serial imitation mechanism. He could have up to 4 attempts at imitation, each of which could be selected in the user interface. The caregiver accepted or rejected Elija's responses by clicking on appropriate buttons. An important aspect of this infant-caregiver interaction was that they could engage in repetitive loops (Fig. 4). The word spoken by the caregiver could be repeated, which sometimes provoked a better response. This could continue until Elija performed an acceptable production, or the caregiver chose to give up and try another word.

### Phonemic transcriptions of the caregiver's responses

To quantify the performance of Elija and his caregivers, we analyzed their interactions during the response and word-teaching experiments. Infant speech is problematic to interpret and analyze but the adult utterances could be readily examined.

Experienced phoneticians created a broad (phonemic) transcription of the caregiver responses, using symbols from the SAMPA inventory [55]. This restricted them to classification in terms of the phonemes of the language they were transcribing or marking utterances as being outside L1. For a given initial motor pattern, several cases were distinguished:

1. A caregiver response that could be straightforwardly coded within a CVC or CVV framework, with at least one V or C and empty slots coded with the symbol ‘,’ (comma).

2. A silent response, which was coded with the symbol ‘#’.

3. A response that could not be transcribed phonemically. (Typically this was an attempt at mimicry by the caregiver.) This was coded as ‘xxx’.

4. A response that was longer than CVC or CVV. From examination, we found that these were cases when the caregiver imputed some precocious linguistic ability to Elija, as if he had produced a progressive phonological idiom. For example, one caregiver responded to Elija's utterances as ‘hello’, on three occasions. This was coded using just the first 3 elements of the response as above.

During data analysis, we analyzed the responses within (1) and (4) in terms of their phonemic transcriptions.

### Archiphoneme consolidations

It is not possible to make a meaningful comparison of the responses of the caregivers at a phonemic level across speakers of different languages since both the nature of segments and segment inventories in any language differ. A further analytical issue is that it is easy to be overwhelmed by the number of phonemic categories that cross-speaker comparisons entail, even within the same language. We therefore grouped phonemes into archiphoneme categories (notated with pipes, e.g. |A|), so that cross-language comparisons could be carried out and comparisons between caregivers presented visually. The relationship between the archiphoneme categories and the phonemes they include are shown in SAMPA notation in Table 1.

**Table 1:** Archiphoneme consolidations for English, German and French.

Archiphoneme	English phonemes	German phonemes	French phonemes
|pb|	/p b/	/p b/	/p b/
|td|	/t d/	/t d/	/t d/
|kg|	/k g/	/k g C x/	/k g/
|tSdZ|	/tS dZ/	/ts tS dZ/	
|?|		/?/	
|fv|	/f v/	/pf f v/	/f v/
|TD|	/T D/	/T D/	
|sz|	/s z/	/s z/	/s z/
|SZ|	/S Z/	/S Z/	/S Z/
|h|	/h/	/h/	/h/
|m|	/m/	/m/	/m/
|n|	/n N/	/n N/	/n N/
|J|			/J/
|R|		/R/	
|r |	/r/		
|l|	/l/	/l/	
|j|	/j/	/j/	
|w|	/w/		/w/
|ie|	/I i e E i: eI I@ e@ jI ji je jE ji: jeI jI@ je@ rI ri re rE ri: reI rI@| re@ lI li le lE li: leI lI@ le@/	/I E i: e: E: jI jE ji: je: jE: rI rE ri: re: rE: lI lE li: le: lE:/	/i e E ji je jE ri re rE li le lE/
|A|	/{A: A aI aU j{jA: jA jaI jaU r{rA: rA raI raU l{lA: lA laI laU/	/a a: aI aU ja ja: jaI jaU ra ra: raI raU la la: laI laU/	/a a∼ A ja ja∼ jA ra ra∼ rA la la∼ lA/
|O|	/Q O O: OI jQ jO jO: jOI rQ rO rO: rOI lQ lO lO: lOI/	/O o: OY jO jo: jOY rO ro: rOY lO lo: lOY/	/o o∼ O jo jo∼ jO ro ro∼ rO lo lo∼ lO/
|UV|	/V U u u: U@ jV jU ju ju: jU@ rV rU ru ru: rU@ lV lU lu lu: lU@/	/Y U u: y: jY jU ju: jy: rY rU ru: ry: lY lU lu: ly:/	/u y ju jy ru ry lu ly/
|&|	/3: 3′ @U @ @′ j3: j3′ j@U j@ j@′ r3: r3′ r@U r@ r@′ l3: l3′ l@U l@ l@′/	/9 2: @ 6 j9 j2: j@ j6 r9 r2: r@ r6 l9 l2: l@ l6/	/e∼ 2 9 9∼ @ je∼ j2 j9 j9∼ j@ re∼ r2 r9 r9∼ r@ le∼ l2 l9 l9∼ l@/

The archiphoneme transcriptions were derived from the phonemic transcriptions, and then separated into their vowel and consonant components so that these could be analyzed separately. That is, the individual components C_1_ V_1_ V_2_ C_2,_ any of which may or may not have been present, were identified.

### Data visualization

After labeling, each subject's experimental data consisted of the presence or absence of an archiphoneme description for each of Elija's 927 utterances.

There was a single labeling dataset for each subject, except for one English speaker for whom there were four datasets. To enable us to quantify how different subjects behaved, and also how one subject behaved in different experimental sessions, we compared the labeling across the relevant datasets. To compare any two datasets, we made pairwise comparisons between the two potentially different labels given to each of Elija's 927 utterances. We did this separately for the C's and the V's.

To make it easier to interpret the results of the comparisons visually, we summed the occurrence of each vowel and consonant archiphoneme across all responses for each subject in the paired comparison, creating two archiphoneme incidence histograms.

We then investigated how the two subjects differed in their particular responses. If both subject responses to a given token were assigned the same archiphoneme label, a ‘same label’ incidence counter was incremented. Differences in labeling were recorded by incrementing an incidence counter assigned to the non-matching archiphoneme pair.

One goal of this study was to assess if subjects with different language backgrounds respond to Elija in a different way. To achieve this we needed to compare responses across different groups of subjects, and not just between individual subjects. To do so, we extended the summing procedure described above over all the multiple pairs of datasets under investigation. Such individual two-session pairwise comparison results and also the multiple group comparison results can be plotted to visualize similarities and differences in individual caregiver's responses.

To generate a more abstract description of group comparisons that could be used for statistical analyses, we summed up the total ‘same’ and ‘different’ archiphoneme responses. This gave a single overall measure of similarity between the compared dataset groups without reference to any specific detail regarding which archiphonemes were involved in the comparisons.

### Statistical analysis of results - Difference of two proportions

To determine the significance of differences between the same response conditions, we used a Z-test to compare the two population proportions. We briefly summarize the calculation of this test statistic below:

Since we had a sufficiently large number of samples in Experiments 1 & 2, that is:



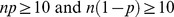
where:


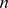
 is the number of samples


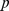
 is the probability of the tested proportion

We calculated the 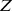-test statistic assuming a normal distribution:
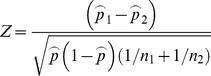
where:
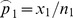





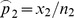





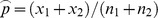
To test the null hypothesis that the two proportions are equal:
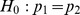
We used a 2-sided decision rule at 3 levels of significance:

For 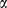 = 0.05 decision rule, 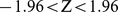


For 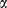 = 0.01 decision rule, 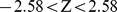


For 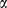 = 0.001 decision rule, 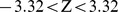


### Bargraph confidence intervals

We calculate the confidence intervals such that:

Lower bound: 
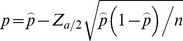


Upper bound: 
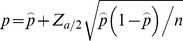


We computed the lower and upper bounds for a confidence value of 95% (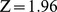)

## Results

### Experiment 1 - Investigating caregiver responses in 3 languages (n = 8)

As babbling commences, interaction with a caregiver can shape an infant's vocal development [56]. To investigate the behavior of caregivers when an infant vocalizes, interaction experiments were run using native speakers of English, French and German playing the role of caregiver. Subjects consisted of 2 English females (E1, E2), and 2 English males (E3, E4-1), a French Canadian female (F1), French female (F2), a German female (G1) and a German male (G2). Each caregiver interacted with a separate (but initially identical) instance of Elija, so that during their experimental session only their own interactions would affect Elija's learning.

Elija's motor patterns and acoustic output are examined in Appendix S2 in File S1. In particular, utterances that were responded to by caregivers are compared against those that were ignored.

### Basic response statistics

We analyzed the interactions between Elija and his caregivers in terms of the consonant and vowel archiphoneme descriptions of the caregiver's responses. First, vowel and consonant occurrence statistics were calculated. Further analysis then examined similarities and differences in archiphoneme components across the subjects as previously described.

**Figure 7:**
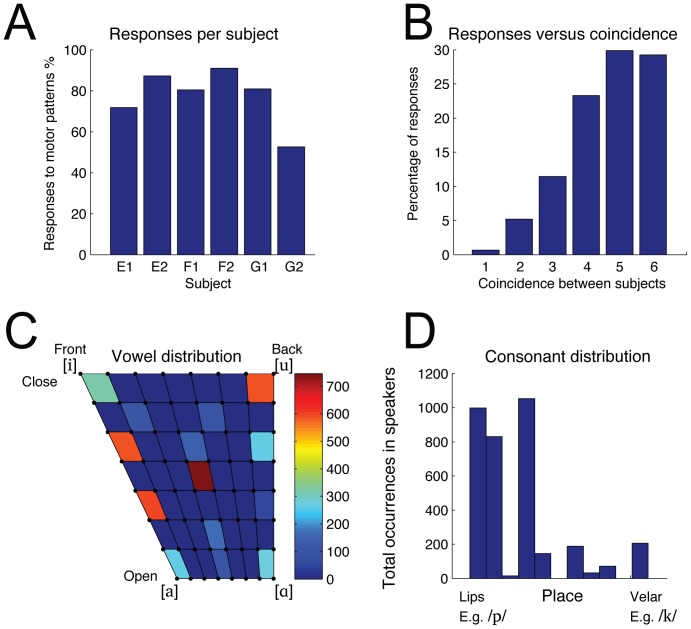
Statistical analysis of the 6-caregiver multilingual response dataset. A Percentage of Elija's motor patterns responded to by each individual caregiver. B Percentage of motor patterns responded to against the number of caregivers that responded to them. C Distribution of vowel qualities plotted on the IPA vowel quadrilateral. The spread of the data shows that the vowel qualities in Elija's utterances as perceived and responded to by the caregivers covered a wide range. D Distribution of the consonantal places of articulation. A wide range of perceived places of articulation were present in Elija's utterances.

Fig. 7 shows analysis of some basic aspects of the response data across the multilingual dataset for 2 subjects in each language (E1, E2, F1, F2, G1, G2).

Fig. 7A shows the percentage of Elija's motor patterns responded to by each individual subject. The value ranged between 53% and 91% with an average of 78%. The spread of responses, even for caregivers within the same language group, indicates that the different subjects used different response criteria.

Fig. 7B shows the percentage of Elija's motor patterns responded to as a function of the number of speakers that responded to them. Note that the total across all subjects sums to 100%. This plot shows that no single motor pattern was ignored by all 6 caregivers.

Fig. 7C is a histogram of the vowel qualities in the caregivers' responses, plotted on the 2-dimensional IPA vowel quadrilateral. Since most responses were reformulations (see below), the spread of the data shows that the vowel qualities in Elija's utterances as perceived and responded to by the caregivers covered a wide range, indicating that the self-organizing vowel discovery process had been effective. Fig. 7D is a complementary analysis of the distribution of the consonantal places of articulation. Again, the perceived places of articulation in Elija's utterances spans the complete range available (from the lips to the velum).

### Transcription-based response analysis

We classified responses as being reformulations, mimicked or idiomatic. A reformulation was a response from a caregiver corresponding to her L1 interpretation of Elija's utterance. A mimicked response was where a caregiver copied the sound shape of Elija's utterance, rather than interpreting it within L1. That is, her response was an acoustic recreation of the utterance. An idiomatic response was when a caregiver credited Elija with having attempted to say something meaningful in L1, and responded with an L1 word or string of words. For example, if she responded to a CVCV from Elija by saying, ”Good morning!”

**Figure 8:**
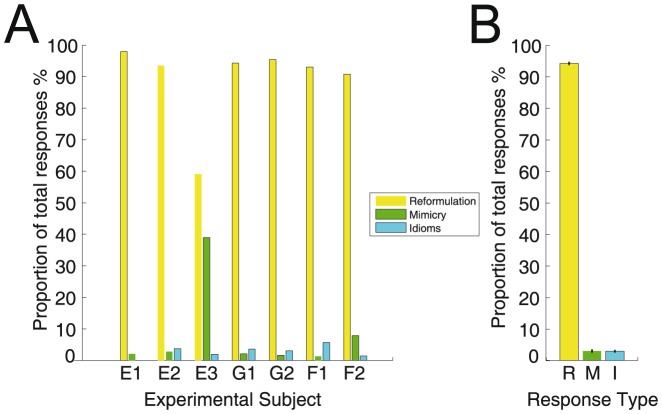
Caregiver response statistics. Responses of different types made by caregivers to Elija's motor patterns are shown as a proportion of total responses. Panel A shows the overall proportions of reformulations (yellow bars), mimicked responses (green bars) and idiomatic responses (blue bars) for all individual subjects. Panel B shows the mean across all subjects with the exception of E3, who was treated as an outlier since he mimicked many more responses than the other caregivers.

Fig. 8 shows the way in which the caregivers responded to Elija's motor pattern repertoire. Panel A displays individual subject data for all the caregivers who were naïve to the purpose of the experiment. This shows the overall proportions of reformulations into L1, mimicked responses and idiomatic responses. Panel B shows the mean across the five subjects who behaved similarly. E3 is being treated here as an outlier since he mimicked many more responses than the other caregivers. This is considered in the Discussion below.

On average over 94% of all responses were reformulations, with an almost equal split between the mimicked and idiomatic responses, which made up the remainder. An idiomatic response is also a source of information about motor pattern/sound value correspondences to a child or Elija in terms of the paradigm for the development of pronunciation that we are investigating. So it can be seen that almost all the caregiver responses were of potential value to Elija for the word learning experiment that followed.

### Visualizing caregiver response across languages

**Figure 9:**
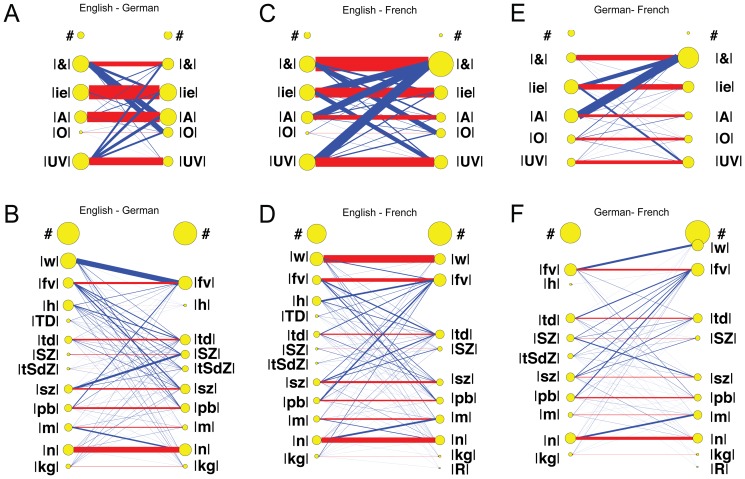
Relationship between English, German and French responses. Summed caregiver response comparisons are shown in terms of their archiphoneme vowel and consonant components. One set of response sessions is represented on the LHS and another set on the RHS of each panel. The area of the yellow nodes represents occurrences of the given phonemic category. Red line width indicates incidence with the same interpretation across sessions; blue line width indicates incidence with a different interpretation across sessions. The 4 English response data sessions are always represented on the LHS and the 2 German and 2 French data sessions on the RHS of each respective panel. A English/German vowel comparisons. B English/German consonant comparisons. C English/French vowel comparisons. D English/French consonant comparisons. E German/French vowel comparisons. F German/French consonant comparisons.

Each response to an Elija utterance could potentially contain consonant and vowel archiphonemes. Pairwise comparisons for archiphoneme categories of first vowels V_1_ and consonants C_1_ were carried out between the responses in the English and German speaker sessions. The English-German pairwise comparisons were then combined to give a single dataset to represent overall English-German group behavior. English-French and German-French comparisons were made in a similar fashion. These comparisons are plotted in Fig. 9.

Panel A shows English/German vowel comparisons and panel B shows English/German consonant comparisons. Panels C and D, and E and F show the same comparisons for English/French, and German/French respectively.

The area of the yellow nodes represents the summed occurrences for all pairwise comparisons of the given archiphoneme category in the responses of the speakers of a given language. It can be seen that there were different numbers of occurrences across the different archiphoneme categories. In all languages, the vowels were fairly uniformly distributed in incidence except for the lower incidence in the |O| category. Consonant incidence was also fairly uniformly distributed except for some lower incidence categories e.g. |TD| and |tSdZ|. The symbol ‘#’ represents incidence when no archiphoneme of type consonant or vowel was found in a particular response.

The summed ‘same label’ incidence, in which motor patterns received the same interpretation across the paired sessions, is plotted using a red line. The summed ‘different label’ incidence, in which motor patterns received a different interpretation across the paired sessions, is plotted using a blue line. In both cases, line width is proportional to incidence numbers.

From Fig. 9A it can be seen that for most of Elija's vowel productions there was reasonable agreement in labeling among English and German caregivers. The main point of disagreement was in the labeling of some responses as |&| by English speakers but as |A| and |O| by German ones.

For the consonants in Fig. 9B, a thick blue line shows that there was a difference in interpretation for motor patterns whose results were heard as |w| by English speakers and |fv| by the German ones. This would be expected, given the absence of/w/in German.

Figs. 9C & 9D show comparisons between the interpretations made by English and French speakers. For the vowels in 9C, it can be seen that a significant proportion of the sounds labeled as |ie|, |A| and |UV| by the English caregivers, were interpreted as |&| by French speakers, presumably reflecting the wider range of vowels that form this category in French.

Figs. 9E & 9F shows the comparisons between the interpretations made by German and French speakers. In the vowels, sounds labeled as |A| by German speakers were often labeled as |&| by French speakers. This suggests that for low and central sounds, the |A| and |&| categories respectively, French and German speakers have different boundaries for categorical perception.

### Experiment 2 - Investigating single caregiver response variability (n = 1)

Experiment 1 showed that there were some differences in how caregivers of English, French and German responded to the same motor patterns. Experiment 2 investigated the similarity in caregiver response within the same single English speaker. To collect the data, E4 performed the response task 4 times following the procedure adopted in Experiment 1. Periods of a week were left between response sessions to reduce the subject remembering Elija's productions from the previous session.

### Visualizing caregiver response across sessions

**Figure 10:**
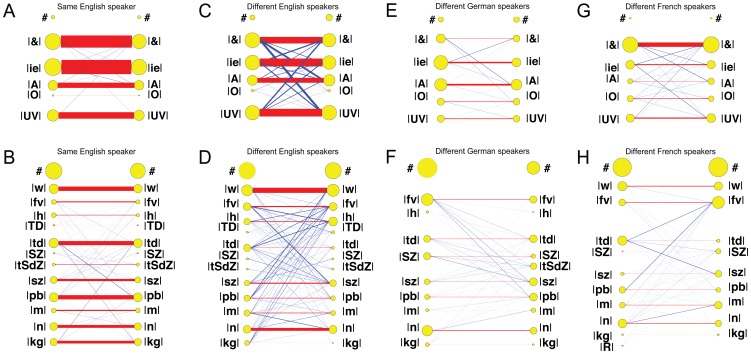
Relationships within English, German and French responses. Results are plotted as in Fig. 9. A & B Vowel and consonant comparisons for a single English speaker over four separate sessions. C & D Vowel and consonant comparisons between four different English speakers. E & F Vowel and consonant comparisons between two different German speakers. G & H Vowel and consonant comparisons between two different French speakers.

Pairwise comparisons for archiphoneme categories of first vowels V_1_ and consonants C_1_ were carried out between all the responses for 4 sessions of this single English speaker. These pairwise comparisons were then summed to give a single dataset to represent single speaker behavior across multiple sessions. These comparisons are plotted in Fig. 10. The vowel and consonant comparisons are shown in Panels A & B respectively.

We also investigated how 4 different English speakers responded to the same motor patterns. The multiple speaker English/English vowel and consonant comparisons are shown in Panels C & D respectively. Similarity between the two German speakers and the two French speakers are shown in Panels E & F and G & H respectively.

The high proportion of red to blue shows that the single English speaker was consistent across sessions, whereas different speakers of the same language exhibited more variety in their interpretation of Elija's utterances.

### Overall similarities across groups

**Figure 11:**
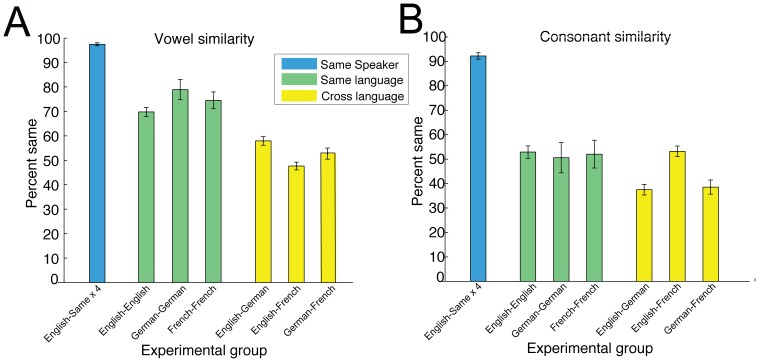
Comparison between caregiver responses. The comparisons are made in terms of their archiphoneme vowel and consonant components. These values correspond to the red lines shown on Figs. 9 and 10. Panels A & B show vowel and consonant response comparisons respectively: similarity within the single English speaker is shown as the blue bar, different speaker similarity for same language groups are shown as green bars, and cross language group similarities are shown as yellow bars. The error bars show 95% confidence intervals.

Fig. 11 shows a plot of the comparisons between caregiver responses for the seven different experimental groups made in terms of the summed archiphoneme vowel and consonant components. These values are the sum of the counts corresponding to the red lines shown on Figs. 9 and 10. Note also that the sum of the blue lines corresponds to the differences in interpretations (which is given by [100% - % same]). We therefore refrain from additionally plotting the percentage difference values to avoid redundancy.

The percentage bars on Fig. 11 correspond to similarities in labeling in the following groups:

- Same English speaker, 4 sessions (English-Same ×4)
- 4 English speakers (English-English)
- 2 German speakers (German-German)
- 2 French speakers (French-French)
- 4 English and 2 German speakers (English-German)
- 4 English and 2 French speakers (English-French)
- 2 German and 2 French speakers (German-French)

We note that the 95% confidence intervals on these plots are generally quite small due to the relatively large number of data counts in each condition, except for the comparisons between the 2 German speakers, and between the 2 French speakers. In these comparisons, there were only 2 speakers in each group and consequently only a single pairwise comparison was carried out. From the Figure it can be seen that the single English speaker was very consistent across sessions in terms of both vowels and consonants. The vowel comparisons for different speakers of the same language groups were more similar than the relevant comparisons across languages groups.

### Multiple speaker comparisons across language groups

We performed Z-tests to compare the differences of the raw vowel and consonant counts data between the selected groups shown on Fig. 11.

To investigate differences in labeling across speakers of different languages, we compared the similarity across speakers within single language groups to the similarity across speakers within different language groups.

### English group comparisons

The ‘same’ proportion for vowels between the different English speakers were significantly different than those between the English-French and English-German comparisons, with p<0.001:

English-English versus English-German, 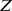 = 9.11787

English-English versus English-French, 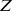 = 17.3888

The ‘same’ proportion of consonants in the different English speakers were significantly different than those in the English-German speaker comparisons with p<0.001

English-English versus English-German, 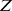 = 8.8926

However, the consonants in the different English speakers were not significantly different from those in the French speaker comparisons, that is, p>0.05

Consonants English-English versus English-French, 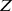 = -0.189129

### German group comparisons

The ‘same’ proportion for vowels between the different German speakers were significantly different than those between the English-German and German-French speakers comparisons, with p<0.001:

German-German versus English-German, 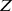 = 7.79693

German-German versus German-French, 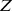 = 9.28878

The proportion of consonants in the different German speakers were significantly different than those in the English-German speaker comparisons and the German-French speaker comparisons, with p<0.001

German-German versus English-German, 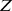 = 3.9623

German-German versus German-French, 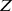 = 3.47732, p<0.001

### French group comparisons

The ‘same’ proportion for vowels between the different French speakers were significantly different than those between the German-French and English-French speakers comparisons, with p<0.001

French-French versus English-French, 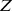 = 12.4145

French-French versus German-French, 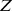 = 9.48962

The proportion of consonants in the different French speakers were significantly different than those in the German-French speaker comparisons with p<0.001

French-French versus German-French, 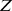 = 4.16795

The proportion of consonants in the different French speakers were not significantly different than those in the English-French speaker comparisons

French-French versus English-French, 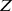 = -0.377895, p>0.05

### Cross language results conclusions

The vowel comparisons between the 4 different English speakers' responses were significantly different from those in the comparisons between the English-German and English-French speakers groups. This was also the case between the 2 different German speakers and the English-German group and German-French group. The 2 different French speakers and the French-German comparisons and English-French comparisons also showed the same effect

These results show that the vowel labeling was more similar within a language group than across language groups. Results for the consonants were not as clear-cut. The consonant labeling was only more similar within a language group than across language groups for the English and German comparisons, and the French and German comparisons. The consonant labeling by English and French speakers was not more consistent within each language group than across them.

The spread of responses within the 4 different English speakers, within the 2 different German speakers and within the 2 different French speakers showed that the caregiver's own individual interpretation played a role in the process. It seems likely that such differences in interpretation arose because Elija's productions were not centered on phonemic categories and therefore a caregiver needed to make an interpretation to determine the appropriate category. This process was subject to their personal biases.

Thus the caregivers showed a systematic bias in the interpretation of Elija's output vowels within the framework of their native languages, with labeling within a language group being significantly more similar than labeling across language groups.

### Evaluating single English speaker consistency

To investigate single speaker consistency, we compared similarity within the single English speaker group to the different English speaker group.

Analysis showed that the ‘same’ proportion of vowels between the 4 repetitions of the single English speaker was significantly different than those between the different English speakers group, with p<0.001:

English-Samex4 versus English-English, 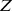 = 26.2974

The ‘same’ proportion of consonants between the 4 repetitions of a single English speaker was also significantly different to the different English speaker group, with p<0.001:

English-Samex4 versus English-English, 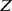 = 24.8201

The statistics shows that the single English speaker was very consistent across 4 different sessions, whereas four different English speakers showed significantly less similarity. Since the multiple repetitions of the single English speaker were significantly more consistent that the labeling made by different speakers of the same language, this indicates that caregivers appear to use personal biases during the labeling procedure.

### Experiment 3 - Learning words in 3 languages by serial imitation (n = 6)

Experiment 3 investigated Elija's ability to learn to pronounce words. Using his acquired ability to parse input speech sounds in terms of the equivalents to his own tokens, the caregivers taught Elija to pronounce some simple words by serial imitation. Elija matched sounds in the new words that were presented to him with sounds he had heard in the first interaction experiment, and used his motor pattern associations to the latter to pronounce the word.

In separate experiments, six (n = 6) subjects speaking three languages (E1, E2, F1, F2, G1, G2) who had previously participated in sound response Experiment 1, once again played the role of caregiver. They were instructed to teach Elija some simple words in their native languages: 219 English, 219 French and 237 German words. The word lists are shown in Appendix S3 in File S1. Each caregiver decided for themselves if they considered their attempt to teach Elija a new word was successful or not – that is, whether his attempt was an acceptable imitation of their word. Overall each caregiver succeeded in teaching him to pronounce between 40 and 72 (mean 55) words.

Experienced phoneticians annotated each caregiver's spoken word data. To analyze Elija's word productions, we used the caregiver's responses corresponding to the motor patterns used by Elija to imitate the word. The latter had been annotated previously for the response comparisons. As before, consonant and vowel archiphoneme components were then extracted. From observation of the interaction process it was apparent that by changing how they spoke the caregivers could sometimes provoke a better response from Elija.

**Figure 12:**
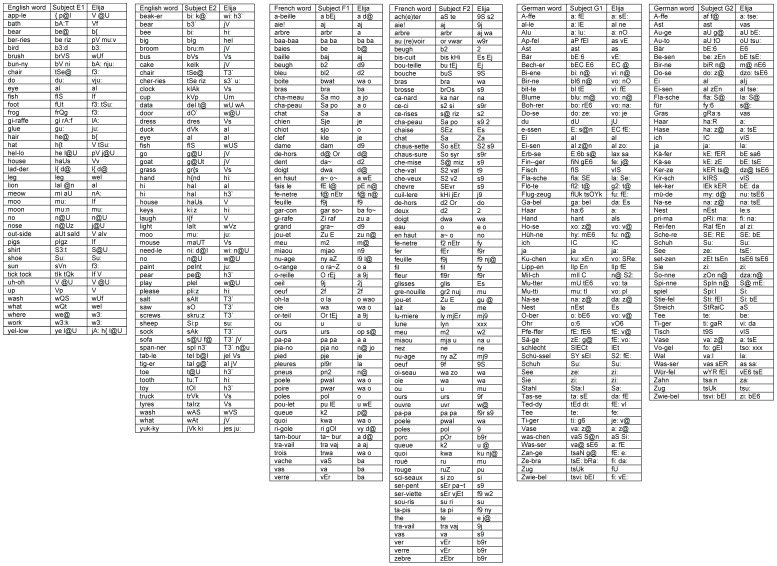
Examples of words learned by Elija. Results for 2 subjects speaking English, French and German are shown for subjects E1 & E2, F1 & F2 and G1 & G2 respectively. The left column specifies the target word, and the middle column is the phonemic transcription of the caregiver's final target production. The right column is the phonemic transcription of the caregiver's reformulations corresponding to Elija's imitations.

Fig. 12 shows the words learned by Elija in the three languages. The results for caregivers speaking English, French and German are shown for subjects E1 & E2, F1 & F2 and G1 & G2 respectively. The left hand column specifies the word orthographically, the middle column is a phonemic transcription of the caregiver's final production and the right hand column is a phonemic transcription of those caregiver responses (reformulations) that Elija recognized in the target word and then used to recall the motor patterns to generate the imitation. Of course, Elija's utterance did not sound like that of an expert speaker since his speech sounds were not as categorically well defined as those of a mature speaker of L1.

To compare the target words produced by the caregivers to the words produced by Elija in response, we compare the archiphoneme representation of the first with that of the second. We transcribed the caregivers' words directly. However, for the reasons described earlier, we do not transcribe Elija vocalizations, but instead use transcriptions of the sounds (reformulations) made by the caregivers that they considered equivalent.

**Figure 13:**
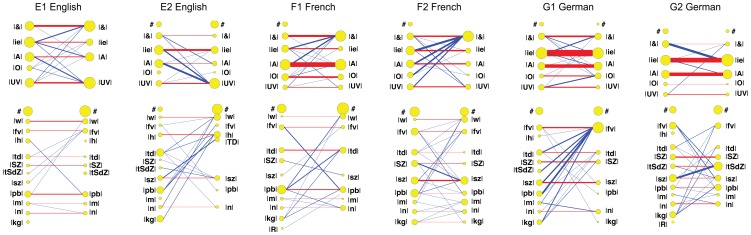
Individual subject word comparisons for English, French and German. Comparisons between archiphoneme representations of caregiver target words and Elija's imitations. Individual speakers are shown in the six panels E1 & E2, F1 & F2 and G1 & G2 respectively. The caregiver target word transcriptions converted to archiphoneme categories are shown on the LHS of each diagram. Elija's imitations were labeled in terms of archiphoneme of the component responses from which they are constructed. These are shown on the RHS of each diagram.

Fig. 13 shows these comparisons (between the speech sounds in the caregivers' word productions and the caregivers' interpretations of the speech sounds in Elija's imitations). The latter were already labeled previously since they were established during the first interaction experiment. The speech sounds were analyzed in terms of first vowels V_1_ and consonants C_1_. The results are presented individually for each of the 6 caregivers. This data is analyzed further in Appendix S4 in File S1.

Sound files of the caregivers' word productions and Ejija's imitated output are available online at https://github.com/HowardLab/Elija-PlosOne-2014. Details of this Online Data repository are described in Appendix S5 in File S1.

## Discussion

### Summary

Using a computer model that starts its development with no speech expertise but general capabilities that are similar to those of an infant, we have shown that for young children to learn to pronounce words, the core element of learning speech sound correspondences need not be an imitative process on the part of the child. Rather than using acoustic matching, as usually assumed, Elija associates his vocal actions with the speech sounds he hears in response to them. This is enabled by mirroring behavior on the part of the caregiver as observed in natural situations and displayed (without being coached) in our experiments. The interaction selectively reinforces Elija's range of potential speech sounds, and the associations he creates from his caregiver's responses allow him to develop an inventory of motor pattern to speech sound correspondences. Thus the behavior of his caregivers enables him to develop a first model of L1 pronunciation.

It was found that when a caregiver found it natural to respond, the unprompted form of the response to a sound that Elija had discovered was almost always a reformulation of Elija's utterance into well-formed sounds of L1 (and occasionally an attempt to mimic his output). The nature of the motor/sound associations was determined by a judgment of sound similarity (or equivalence) made by the *caregiver* rather than by Elija. This is a major point of difference between our account and those of acoustic matching theories of speech sound development.

In our study, separate instances of Elija learnt to pronounce simple words in English, French and German. Our account of this aspect of speech acquisition is demonstrated to be both effective and language independent. Elija is the first model to achieve this (1) using natural and well attested social interactions, (2) given initial perceptive, productive and associative mechanisms that are clearly no greater than those of a human infant, and (3) given no precocious phonetic skill in judging similarity between his own and caregiver speech.

### Three stage operation

In a real infant, the three stages of learning modeled separately in Elija would overlap. In this study, the stages - unsupervised sound discovery, the first sound response experiments and the final word imitation experiments - were kept separate for three reasons. Firstly, it enabled interaction time with caregivers to be kept within practical limits. Secondly, all caregivers heard the same sounds, so that comparisons could be made across their responses. Thirdly, in the two interactive stages it avoided both parties (Elija and caregiver) needing to interpret the nature of a given interaction, since this was unambiguously fixed within the context of each of the experiments: either involving the caregiver in responses to an utterance or in word-teaching.

In principle Elija could be run with the stages overlapping and informing each other. This would model the fact that initial motor patterns can form the starting points for later motor pattern discovery and that motor patterns can change over time. Such operation would require additional mechanisms within the Elija model, to detect and act on the context of a given interaction. That is, Elija would need to interpret the intent of the caregiver and thereby use any given interaction in an appropriate fashion.

### Response results

To quantify caregiver behavior during the interaction experiments we did not attempt to transcribe Elija's production data directly, since this is well known to be problematic in the study of speech development [30]. Instead we analyzed Elija's productions in terms of their corresponding caregiver responses.

Our results show that subjects found it natural to respond to most of the motor patterns Elija produced, and almost always did so with responses that were well formed in L1 and therefore of value to Elija in the word learning experiment that followed.

One can ask why reformulation is the preferred response of caregivers. One reason may be because caregivers credit infants with ‘fully human powers of social responsiveness: with wishes, intentions and feelings which can be communicated,’ [57]. So an L1 response can feel natural even if the stimulus from the infant is not yet actually linguistic.

Furthermore, it is effectively effortless for a highly practiced speaker of L1 to produce speech sounds, so whenever this is felt to be appropriate a reformulation will be an easy response to make. Whatever the reasons, it is interesting to note that Elija's caregivers did behave in this way towards him despite Elija not being their own child and not even being a real child. The motivation in adults to reformulate an infant's output appears to be quite powerful.

### Comparing our results with human studies

Studies of natural caregiver-infant dyads relevant to these experiments with Elija have covered the ages 2 to 4 months (17 pairs) [58], 2 to 6 months (15 pairs) [59], 4 to 11 months (8 pairs) [60], 9 to 19 months (6 pairs) [61], and 12 to 21 months (3 pairs) [62]. The behaviors of both caregivers and infants that these studies report are consistent with the Elija model of how children learn to solve the correspondence problem by the caregiver imitation of infant vocalizations. Such caregiver imitations are reported to be ubiquitous, to occur with high frequency and to be more common than infant imitations of caregiver utterances. We note that this well-documented and widely recognized phenomenon is not given any role in current speech development theory, including in acoustic matching accounts, but forms the basis of the Elija model derived from Gattegno's observations and theorizing [27] as well as the work of the Asada group [24].

Pawlby's data [60] may cast a light on the behavior of subject E3 in our study, who was an outlier compared to our other subjects, reformulating around 60% of the motor patterns he responded to, and mimicking about 40%. Pawlby analyzed imitative exchanges across five modalities, including her Group III, speech sounds (vowel-like, early consonantal and late consonantal sounds), and Group IV, non-speech sounds (whimpers, laughs, raspberries, etc.). In infant-mother sequences, Pawlby recorded 625 instances of mothers imitating infant speech sounds (Group III) and 261 instances of mothers imitating non-speech sounds (Group IV). (There were many fewer mother-infant sequences: infants imitated 60 speech sounds produced by their mothers and 16 non-speech sounds.)

If we equate the reformulating and mimicking responses of our subject E3 to Pawlby's Groups III and IV respectively, his behavior is within the range she observed in her eight dyads. It may be, therefore, that he was conceiving Elija to be around the age of infant that Pawlby tested, and responded appropriately, while the other subjects conceived Elija to be older, granting him more capacity for linguistic communication and responding appropriately to this.

Pawlby's extensive study enables a comparison to be made between imitation of vocal acts and other acts. She found a commonality across all forms of imitation, vocal and non-vocal, and, in line with all the other studies referenced above, found that, ”the mother's imitation of the infant's acts is a much more frequent phenomenon than the infant's imitation of his mother's acts. It is the infant who is more likely to initiate the sequence and his mother respond by imitating.“ Overall Pawlby interpreted her findings as indicating that, ”the whole process by which the infant comes to imitate his mother in a clearly intentional way is rooted in the initial readiness of the mother to imitate her own infant.“ The Elija model is an instance of this learning paradigm.

### Differences between caregiver behaviors

During the mirroring process, some caregivers responded more frequently than others, demonstrating that there is variability between individuals in their threshold criterion for a response.

The (archiphoneme) vowel components in the responses of caregivers were more often similar to those of the other speakers within a given language group than to those of other language groups. For example, the English caregivers' responses to Elija's sounds were more consistently similar to those of other English speakers in vowel quality than to those made by the German and French caregivers. Thus at least for vowel qualities, caregivers showed a significant systematic bias to interpret Elija's output within the framework of their L1.

The vowel and consonant qualities in the responses made by the same English caregiver over 4 separate sessions were very similar, motor pattern by motor pattern. However, there was significantly more spread of interpretations of a given motor pattern across different caregivers of English. This suggests that the differences in responses within different speakers of the same language group may have arisen from different systematic interpretations, rather than from an underlying noisy process. Categorical perception of speech may explain this, since Elija's productions were not in any way limited to being good L1 exemplars. Consequently, many sounds would have been ascribed by a caregiver to what he or she considered to be a ‘good enough’ category. Their judgments on this would certainly have varied.

### Learning words by serial imitation

In the final experiment, word learning was carried out using a distinct imitation stage that assumed that a motor pattern/sound response repertoire had already been established. Running this stage separately from the response stage was efficient from an experimental perspective, since it meant that neither Elija nor the caregiver needed to interpret the context of the interactions. The six caregivers succeeded in teaching Elija to pronounce an average of 55 typical first words in their languages.

The serial imitation process relied on Elija's recognizer evaluating the caregiver's utterances in terms of sounds that Elija had heard before. This was implemented using dynamic time warping (DTW). However, high performance speech sound recognition on the basis of limited training data is hard to achieve without *a priori* knowledge. Elija's recognition performance was therefore lower than we would expect a human infant to be able to achieve. This occasionally led to some inappropriate interpretations of a caregiver's speech. However, although Elija sometimes made such errors, the caregiver could also correct these as she could prompt him for another attempt at recognition or even speak again until he generated an appropriate response (see Fig. 4). Analogous behaviors on the part of caregivers are seen in natural settings.

Elija did not always generate imitation forms that closely matched his caregiver, since his motor pattern repertoire was not sufficient to do so. However he achieved a level of performance that appears comparable to that of young children aged between one and two years.

### The reformulation phenomenon

Reformulation was the key source of information used by Elija to learn to pronounce and then to learn the pronunciation of words [28]. In our account, an infant learns about the linguistic significance of his utterances (primarily conceived by him as motor patterns) through their reformulation by his caregiver. The ‘mirrored’ structure of such an interaction is already familiar to him from earlier development, where a caregiver is believed to serve as an affective mirror for a young infant [63]. This sometimes involves the caregiver reflecting the surface characteristics of his behavior but increasingly [64] what she reflects is an expression of her interpretation of his inner state (so-called ‘affect attunement’ [65]). In a vocal context, a mimicked response is analogous to the former, and a reformulation to the latter. When they reformulated his vocal actions, Elija's caregivers acted as a phonological mirror, presenting him with what they judged to be L1 equivalents of certain motor patterns. This solution to the correspondence problem then allowed Elija to learn words by serial imitation of their constituent speech sounds.

### Supporting evidence and wider implications

Several converging lines of research are consistent with the Elija model. Gattegno reported naturalistic observations of infants learning to speak in support of his proposal that infants do not learn to pronounce by imitation, but rather by experiments that are evaluated and rewarded by their caregivers [27]. Messum [8] described other experimental and theoretical support for this alternative paradigm.

More recently, speech feedback alteration studies have provided experimental evidence of a lack of acoustic self-regulation of speech output by young infants [9], and even by some adults [10]. These observations are clearly in conflict with the current assumption that imitation is the mechanism by which infants learn to pronounce the speech sounds that form the elements from which words are made up [11]. The idea that there is an absence of self-regulation in speech production in young infants is, however, consistent with a well-known anomaly in child speech, the ‘fis/fish’ phenomena [12], [13]. Here, a child pronounces ”fish” as ”fis”, and when questioned as to why he did so, insists firstly that he can hear the distinction in the two forms made by the adult and secondly that he did not say the incorrect form himself. This behavior cannot be explained satisfactorily under imitative accounts of speech acquisition that require the infant to perform acoustic matching, but is consistent with the Elija model and the lack of self-regulation seen in infants. Clearly if an infant does not or cannot self-regulate on the basis of their acoustic output, they will be unaware of its acoustic form and that their pronunciation is incorrect. This is what is observed.

Support for Gattegno's alternative paradigm in speech development also comes from the demonstration of non-vocal tutoring of young male cowbirds learning to sing by non-singing females [66]. Similarly, in human studies, a number of experiments with infants have shown that caregiver behavior is perceived and used by young learners to generate more advanced forms of vocalization [56].

One of the most basic, longstanding questions about speech is whether it is represented in the brain primarily as a motor or as an acoustic phenomenon: is it ‘gestures made audible’ [67] or an acoustic code? Our proposal escapes the current terms in which this debate is framed: rather than a simple mechanism (imitation) leading to a complex and controversial neural organization of whatever kind, we propose that a learning mechanism involving mirroring by a social partner leads to a straightforward representation that is inherently perceptuo-motor. In this process, neither production nor perception is more primitive than the other. A number of longstanding problems in speech are resolved by this understanding [8].

## Conclusions

It is clearly the case that children learn the pronunciation of words by imitation. However, the mechanism by which a child learns to pronounce – learns, that is, to produce speech sounds that are taken by his listeners to be equivalent to those in L1 - may or may not be imitative. The assumption that this mechanism is an imitative, auditory ‘match to target’ process performed by the child underlies theory and practice in the speech sciences, but is unexamined theoretically and unsupported experimentally.

Elija tests an alternative mechanism for how children might learn to pronounce. Our results demonstrate two important aspects of this. First, that the social learning paradigm involved allows a computational model endowed with capacities that are no greater than those of a human infant to progress from an initial state of no knowledge about speech to the pronunciation of first words. Elija did this without imitating the sound qualities of his caregivers' speech. Second, that in interacting with Elija, human caregivers naturally do what is needed for his development, and what would be needed for human children to develop L1 pronunciation within the paradigm we have described.

## Supporting Information

File S1 **Appendixes S1–S5.**
(PDF)

Figure S1 (TIF)

Figure S2 (TIF)
