# Supplementary material for: Learning to Pronounce First Words in Three Languages: An Investigation of Caregiver and Infant Behavior Using a Computational Model of an Infant
Source: PLoS One. 2014 Oct 21;9(10):e110334. doi: 10.1371/journal.pone.0110334 (PMC4204867; doi:10.1371/journal.pone.0110334)
Supplement: File S1 — Appendixes S1–S5. (PDF) [file pone.0110334.s001.pdf]

Appendix S1: Extended methods of the Elija computational model of an infant

We model an infant as a computational agent, Elija, who has no *a priori* articulatory or perceptual knowledge of speech [1]. The main features of Elija's motor system are shown in Fig. 5A. Elija has a speech production capability based on a modified Maeda articulatory synthesizer [2,3]. This is driven by a motor system in which representations of motor actions are akin to the gestural score used in the Task Dynamics model [4]. A motor pattern is a sequence of articulatory targets for the synthesizer's control parameters. A controller assumes that the articulator movements follow 2nd order critically damped trajectories and interpolates between these targets. The resulting sequences of time-varying parameter vectors drive the synthesizer. This can lead to acoustic output played out via a loudspeaker.

A schematic of Elija's perceptive system is shown in Fig. 5B. Elija's hearing system receives input from a Rode Podcaster USB microphone. Autocorrelation analysis is applied directly to the input waveform to estimate the fundamental frequency F0. An auditory filter bank provides initial pre-processing of the input [5]. Our implementation is based on the gammatone-like spectrograms implemented by Ellis [6].

Analysis of Elija's own acoustic output is carried out directly on the digitized signal from the synthesizer although in principle this could also be achieved by passing acoustic output back from the loudspeaker via the microphone.

We note here that the potential for bone-conducted sound to impair an infant's ability to compare his own production to that of his caregivers is a potential problem for acoustic matching theories, as discussed in [7], but is not a problem with the Elija paradigm. In the sound discovery process, Elija uses a measure of acoustic diversity, with motor, tactile and acoustic metrics. This partly uses acoustic comparisons of Elija's output with his former output. However this constitutes only basic spectral comparison. The mechanism would operate similarly in the presence of bone-conducted speech.

Further processing estimates signal salience, which is used as a component in Elija's reward mechanism. Pre-processed input can be recorded in auditory memory and also compared against past memories using a speech sound recognizer that is based on Dynamic Time Warping (DTW) [8]. This enables Elija to discriminate different speech sounds.

Maeda articulatory synthesizer

Elija has a vocal apparatus based on the Maeda articulatory synthesizer [2,3] and we include a short review of the Maeda model here for the convenience of the reader. The model represents the cross sectional profile of the vocal tract in 2-dimensions along the mid-sagittal plane. The parameters in the model were

estimated by Maeda using factor analysis of an x-ray dataset consisting of cine-radiographic vocal tract profiles and labiofilm frontal lip shape recordings of 2 female French speakers producing 10 French sentences. The images were recorded at 50 frames per second and in total there about 1000 frames of data were analyzed. The vocal tract was divided up into 3 sections – lip opening, principal vocal tract and pharynx. The principal vocal tract was measured in semi-polar coordinates, the lips by an elliptical opening and the larynx by its height. A jaw model [9] was then invoked to explain the dataset in terms of six parameters: jaw, tongue-body, tongue-tip, lip height, lip width and larynx height. Vocal tract shape was assumed to arise from a linear combination of the state of these elementary articulators and a directed factor analysis was used to describe vocal tract shape in terms of these parameters. This method allowed the contribution of a particular elementary articulator to be subtracted from the dataset using linear regression, making it possible to explain the input data in terms of the pre-defined elementary articulators. This would not be the case if standard factor analysis had been used, in which case there would have been no simple interpretation of the action of the factors. The contributions of control parameters were subtracted in a specific order to find orthogonal parameters. Thus - starting with jaw height - jaw, lip and tongue control parameters were estimated.

In our implementation of the Maeda articulatory synthesizer [2,3], ten parameters are used to control the vocal apparatus, the first seven being articulatory: P1 Jaw position, P2 Tongue dorsum position, P3 Tongue dorsum shape, P4 Tongue apex position, P5 Lip height (aperture), P6 Lip protrusion, P7 Larynx height. In addition, an LF voice source model was added to give control over a voiced excitation model [10]. (LF, named after the authors Liljencrants and Fant, is a four-parameter model of glottal flow.) This makes use of two additional parameters: P8 Glottal area, and P9 Fundamental frequency. In the original VTCALCS implementation a velo-pharyngeal port was added to the basic model and its opening can also be controlled using parameter P10 Nasality. Thus the Maeda synthesizer enabled Elija to produce both oral and nasal sounds. After the vocal tract profile is specified by the elementary articulator parameters, an equivalent digital filter is computed and used to filter the excitation from the voice source and other noise sources. Fricatives are simulated in the model by injecting noise at locations in the vocal tract where turbulent airflow is predicted.

In our experiments, the synthesizer operated with an output-sampling rate of 24 kHz. To approximate an infant vocal tract adequately for the purposes of these experiments, the model's default physical dimensions, which originally reflected the sizing of an adult female vocal tract, were scaled down by a factor of 0.8. (We note that there are other differences between adult and infant vocal tracts. For example, this scaling does not reflect some other differences in the size of the pharynx [11]).

Similarly, the mid-range of the fundamental frequency was shifted from 210 Hz to 400 Hz. We added proprioceptive feedback of lip and tongue contact, which was

generated at times when the vocal tract tube cross-sectional area reached zero. Elija was implemented in C++ and all other analyses were written in Matlab (Mathworks Inc, Natick MA, USA) running on a PC. Acoustic output was played to the caregiver from the PC's inboard DAC output via a pair of active loudspeakers.

Modeling motor patterns and articulator dynamics

As in a previous implementation of Elija [1], motor actions were modeled in a way akin to the gestural score used in the Task Dynamics model [4] and movement of Elija's articulators between targets was implemented by assuming 2nd order dynamics that follow critically damped trajectories [12]. In this work we extend our former approach and the dynamic properties of different vocal tract articulators are now no longer all grouped together. Rather they are given individual properties (see below). We note that other approximations to articulator movements could also be made, e.g. using a minimum jerk trajectory, which is often used to describe human arm movements [13].

In Elija, a motor pattern can be a sequence of up to three different sub-patterns. Each sub-pattern specifies parameters needed to control the vocal apparatus and contains a 10-element target vector, a 10-element starting time vector and a 10-element duration time vector specifying how long a target is maintained. There is also a single overall transition speed scaling parameter β. Thus each sub-pattern consists of 31 elements.

Each component target vector gives rise to movement of the articulators from their current state towards their new target values. As stated above, such articulator movement follows a critically damped trajectory, leading to articulator movement towards their targets without overshoot [15]. We compute the trajectory of each control parameter using the equation:

$$x(t) = x_e + (x_s - x_e)(1 + \beta t)e^{-\beta t}$$

Where $x(t)$ is the parameter value at time t, x_s is the starting point, x_e is the end point (target value), the constant β is given by the relation $\beta^2 = k/m$, where k is the spring constant and m is the associated mass of the dynamical system.

The value of β associated with the different vocal tract articulator parameters is matched to their dynamic properties. For movements of the articulators during vocalic, sonorant and fricative sound generation, a value of β = 40 is used, since it matches typical human articulation speeds well. However, during plosive sound generation transitions are much faster due to the rapid release of air pressure at the point of vocal tract closure. To account for this phenomenon, transitions following closure have their associated β value increased to 160. This leads to the generation of more realistic plosive sounds

Unsupervised sound discovery

Elija's discovery of sound-generating motor patterns under developmentally plausible influences is formulated as an optimization problem that operates without caregiver involvement, and is an extension of previous work [14]. The modeling of autonomous exploration has recently become an area of interest for several researchers, including those working in the field of developmental robotics [15-20]. We note that Elija uses both intrinsic and extrinsic reinforcement, as described by Warlaumont [21], during his sound discovery and refinement process.

The objective function

Elija uses rewarded exploration of the vocal tract parameters to find motor patterns that generate vocal actions. This discovery process is formulated as an optimization problem. Optimization is a computational technique that can find the set of parameters of a function that specify its maximum (or minimum) value. Simple gradient ascent (hill climbing) is an iterative process, in which steps are taken in the direction of the gradient. In Newton's method, a Taylor expansion is used in the estimation of the steps needed, which makes use of the curvature of the objective function. This involves computing the second derivative, or Hessian, of the objective function. For computational reasons, quasi-Newton optimization algorithms are often used in practice, which avoids directly computing such second derivatives. In our experiments the parameters to be optimized are those that define the motor patterns, and we use quasi-Newton gradient descent to find values, which maximize their associated objective function or 'reward', as described below.

Computing reward

In our model, the objective function for the optimization of motor patterns includes terms that encourage salience and diversity and discourage motor effort. In addition, we now include a term that discourages the discovery of 'sensitive' motor patterns, as explained below. The continuous scalar reward value R computed in objective function of the algorithm is given by;

$$R = \sum (salience + diversity - effort - sensitivity)$$

Salience

The salience term encourages Elija to find motor patterns that generate sensory consequences. Sensory salience was estimated by combining several components: averaged weighted low and weighted high frequency power over the duration of the motor pattern and the average touch signal.

Specifically, we compute a weighted sum of speech power, ratio of low to high frequency power (above and below 6 kHz), ratio of high to low frequency power (above and below 6 kHz) and high pass filtered touch contact (frequency cut-off =

1 Hz). Second order Butterworth filters were used to implement all the low and high pass filters. We compute salience as:

$$salience = W_{ap}.Power_a + W_t.Touch + W_{hflf}.Power_{hflf} + W_{lfhf}.Power_{lfhf}$$

where
W_{ap} represents the weighting term for acoustic power
W_t represents the weighting term for touch
W_{hflf} represents the weighting term for the ratio of high frequency power to low frequency power,

The individual terms for acoustic power, touch and spectral balance are computed by averaging the time waveforms for these quantities over the length of each vocal action.

We assume that a human infant can and does selectively focus his attention on these different aspects of sensory feedback. Elija does so by changing the relative contribution of the components of salience. Attending to acoustic power at lower frequencies will favor the discovery of configurations that lead to vowel production, while attending to acoustic output with a dominant high frequency component will favor the discovery of fricatives. Attending to touch will favor configurations used in consonants, such as where the lips are closed or the tongue makes contact with the teeth or the roof of the mouth.

Pattern Diversity

The diversity term is included in the objective function to encourage the discovery of a range of motor patterns that lead to different sensory consequences. That is, it encourages the discovery of novel patterns that are different from the previous ones found. Diversity was computed as the weighted sum of three components in acoustic, tactile and motor pattern space. In each of these spaces, the minimum distance arising from the current motor pattern to all previous motor patterns was calculated. The weighting affected the class of motor patterns discovered. A strong tactile weighting biased the optimization to the discovery of distinct plosive articulations, whereas a strong acoustic weighting biased the optimization to the discovery of acoustically distinct vocalic and fricative sounds. We note that such explicit weighting is not strictly necessary, since the diversity term will by its very nature result in active exploration. However its inclusion does speed up the computational process. We compute diversity as:

$$diversity = W_{mdp}.diversity_{motor} + W_{td}.diversity_{touch} + W_{sd}.diversity_{acoustic}$$

where

W_{mdp} represents the weighting term for motor diversity term
W_{td} represents the weighting term for tactile diversity term
W_{sd} represents the weighting term for sensory diversity term
and

$$diversity_{motor} = \min_{all\ patters}[currentMotorPattern - pastMotorPattern]$$
$$diversity_{touch} = \min_{all\ patters}[currentTactileData - pastTactileData]$$
$$diversity_{acoustic} = \min_{all\ patters}[currentAcousticData - pastAcousticData]$$

where the difference from the current motor pattern and its tactile and acoustic sensory consequences are computed for each of the previously discovered motor pattern and their tactile and acoustic sensory consequences.

Effort

The effort required to execute the motor pattern makes a negative contribution to the objective function. Effort was determined by a combination of the cost of movement and the loudness of the voiced excitation. The cost of movement was calculated as the weighted sum of articulator speeds over the duration of the motor pattern. Loudness of the voiced excitation was estimated by summing the voicing contribution to Maeda parameter P8 over the duration of the motor pattern. The effort term is important because if no penalty is included for voicing loudness, the optimization generally finds a solution with the voicing parameter set to maximum, because this always maximizes sensory salience.

Thus Effort is given by:

$$effort = W_{ae}.effort_{articulatory} + W_{ve}.effort_{voicing}$$

where

W_{ae} represents the weighting term for articulator effort and
W_{ve} represents the weighting term for voicing effort

We note that the effort term could be enhanced, for example by incorporating ‘toil’ (relating to the deformation of the vocal tract) as defined by Yoshikawa et al [22].

Sensitivity

A sensitivity term is included in the objective function to penalize the discovery of motor patterns that create sounds that can only be generated by very accurate articulations. More specifically, motor pattern sensitivity relates to how much the acoustic output of a given articulation changes when the motor pattern is subject to local perturbations:

Sensitivity = (change in acoustic output) / (change in articulatory targets)

Sensitivity issues affect the discovery of vowels. Given that some variability is found in speech production and is a feature of the learning process, insensitive articulations will more reliably lead to an acceptable intended acoustic output than sensitive ones. There is reason to believe that very sensitive articulator configurations are not utilized in speech production, as addressed in Steven’s Quantal Theory [23] and Gunnilstam’s Theory of Local Linearity [24]. Both hypothesize that preferred regions of articulation in speech production exist and

that there are, for example, regions of articulator space that provide a natural location for vowel sounds. The sensitivity of the acoustic realization of a given motor pattern was computed by first individually positively perturbing the parameters P1 to P5. A perturbation corresponding to 5% of the full parameter range was used (i.e. a value of 0.1 was added to each Maeda parameter). All other parameters were set to constant values across all motor pattern vectors to avoid added variability in acoustic output. The output time waveforms for the unperturbed motor pattern and for each of the 5 perturbed motor patterns were generated using the Maeda synthesizer and were then analyzed using the auditory filter bank. The distance between the auditory representation of each perturbed motor pattern and that of the unperturbed pattern was computed. The overall sensitivity for the given motor pattern was then taken as the square root of the sum of squares of the 5 components. The perturbed patterns were only used to assess the sensitivity of the pattern under investigation and were not stored in memory.

Running motor pattern discovery

In the Elija model, motor pattern discovery starts by setting the elements of the motor pattern to random values drawn from a uniform distribution over their valid range (-1 to 1). Motor pattern solutions are then found using 3 iterations of a Quasi-Newton gradient descent algorithm, as implemented by the Matlab function fmincon (which finds a constrained minimum).

Since this study investigated sound and subsequent word learning, several steps were employed to ensure that Elija discovered a wide range of suitable motor patterns within a reasonable time. Using single target motor patterns, separate optimization runs were employed with an emphasis on low frequency power (for vowels), high frequency power (for fricatives) and touch (for plosives). To increase the variety of sounds, voicing was explicitly enabled or disabled in each plosive and fricative articulation (that is, this operation was not carried out automatically by the optimization procedure). Similarly, closures were generated with or without opening of the velo-pharyngeal port, creating nasals or plosives respectively. We note that during motor pattern discovery active learning was always present. Therefore, although the *a priori* biasing was used to reduce exploration times, if the motor pattern discovery process had been allowed to run for long enough it would have found a comparable final set of consonants and vowels autonomously, without making such interventions, as was achieved in our previous study [1].

Consolidation of motor patterns

To limit the overall number of motor patterns, clustering was used to reduce the occurrence of articulations that were similar. Such clustering maintained variety, but limited redundancy and ensured that there was no subsequent combinatorial explosion of C and V configurations when sequences were generated (see

below). The clustering of plosive configurations was performed directly on motor patterns using a standard K-means algorithm. Vocalic and fricative sounds were clustered acoustically using a modified version of the same algorithm, using dynamic time warping (DTW) as its metric of similarity [1]. The total number of motor pattern clusters and categories were set by hand to limit their number. Again we note that clustering would be unnecessary if long interaction times with caregivers were acceptable. Ideally, all the raw motor patterns discovered by the optimization search would have been used and evaluated by the caregiver, but this would have required much longer periods of interaction.

The number of vocalic sounds discovered was limited to 15, the number of plosives was limited to 15 and the number of fricatives limited to 10. As a result, the subsequent interaction experiments could be carried out within 2 - 3 hours per caregiver.

Implementation of pattern clustering

As described above, after Elija has acquired a set of motor patterns in an experimental run he uses clustering to consolidate them. Elija can consolidate speech utterances either on the basis of their motor properties or acoustic properties. For the latter, the utterance is analyzed using an auditory filter bank.

Motor patterns are clustered directly using a standard K-means algorithm, as available in Matlab. For acoustic clustering of utterances, which will vary in length (different utterances from Elija will typically have different time durations, as will the caregiver's utterances), the standard K-means algorithm is not appropriate, since it requires a fixed pattern length (see the K-means implementation in NETLAB for further details [25]). Therefore we perform clustering using a modified version of the standard algorithm, which we call DTW K-means. This is similar to the standard K-means algorithm except that 1) it represents a cluster using the best exemplar rather than its mean and 2) it uses a DTW distance metric. It operates in two steps. Let us assume we have already decided on the number of clusters, K. First the algorithm randomly chooses a best exemplar pattern to define each of the K clusters. It then begins an iterative loop. It processes each utterance in the dataset, assigning them to their nearest cluster exemplar. In standard K-means, a Euclidian distance metric is often used to directly compute distance. However, in the DTW K-means algorithm, dynamic time warping is used to determine the distance between utterances (as described in section 3.12). After all utterances have been assigned to a cluster, we then use all the utterances within each cluster to re-compute the best exemplar, where this is defined as the utterance that is on average closest to all other utterances. It is found simply by adding up the distances to all other utterances for each utterance in turn, and choosing the utterance with the minimum summed distance. Then we once again assign each utterance to the closest exemplar. The assignment/re-computation process is repeated until no further change of assignment occurs.

Motor and sensory memory

As motor patterns are discovered, they are recorded in Elija's current motor memory. When Elija uses a vocal action to generate a speech-like sound to which his caregiver responds, her corresponding acoustic response is retained in current sensory memory. In addition, an association is formed between these motor and sensory patterns, which is also retained during clustering. Motor patterns that generate no response are discarded.

Expanding motor pattern variety

By concatenating the simple motor patterns discovered by the optimization procedure, Elija can generate more complex utterances that are potential speech sounds. Single articulations were combined to generate VVs (sounding similar to true diphthongs), CVs, CVVs and VCs. More specifically, Elija generated CV (C_vV, C_uV, F_vV, F_uV, NV), VC (VC_v, VC_u, VF_v, VF_u, VN) and VV tokens, where N = voiced nasal consonant, C_v = voiced consonant, C_u = unvoiced consonant, F_v = voiced fricative, F_u = unvoiced fricative. Longer sequences were in principle possible, but not used in the current study. Again we note that the combination of simple motor patterns into complex motor patterns was only performed to reduce the time needed to discover motor patterns. If the motor pattern discovery process had been allowed to run longer and to find multiple target motor patterns, the complex motor pattern discovery process could operate fully autonomously as in our previous study [1].

After the authors removed implausible sounds by hand (for example, synthesizer artifacts such as clicks), Elija had discovered 927 motor patterns, which could be used for the first response experiments.

Implementing utterance recognition

Elija has no *a priori* phonetic or phonological knowledge but he must learn to discriminate sounds in his environment.

To implement this mechanism, Elija used a template-based dynamic time warping (DTW) recognizer [26], running with an auditory gamma tone filter bank front-end [5]. The DTW recognizer uses the caregiver's responses as its sound templates.

This algorithm aligns and locally warps the input speech utterances to account for differences in timing between them. It compares each frame in the input data with the corresponding ones in a set of reference templates that comprise the vocabulary of the recognizer, and returns a metric of similarity for each. By using dynamic programming (DP), this procedure can be computed efficiently. DP has formed the basis for many speech recognition systems [27]. The implementation of the DP used in our experiments was due to Ellis [8]. Although this algorithm was originally used for music recognition [26], it is equally suitable for speech

recognition since the underlying DP algorithm required is the same in both cases. As mentioned above, the DTW algorithm is also used as the similarity metric in the DTW K-means algorithm.

Since words could contain several basic speech sounds concatenated together, a segmentation mechanism was used to present them individually to the template-based recognizer. This required that the caregiver spoke with pauses between syllables.

Recognizing caregiver sounds

A two-stage procedure was used to recognize caregiver reformulations. This identifies the category of an input sound produced by the caregiver based on acoustic similarity and then the best matching sound within that category. This first required the caregiver reformulations to be partitioned into 100 clusters, a value chosen by experimentation. This was performed using the DTW K-means algorithm described above. The associations with vocal motor patterns were maintained during clustering, so that identification of a reformulation also identified Elija's corresponding motor pattern.

During sound recognition, the DTW recognizer first uses the best exemplars in each cluster as the templates to identify the sound category. The recognizer then uses the members of the best category as templates, to identify the best specific matching sound.

Experiments

The first experiment investigated caregiver responses in three different languages using all 8 subjects. We examined variability of responses within the speakers of the same language. The second experiment investigated the variability of the responses from a single English speaker over 4 sessions. The third experiment investigated word learning by Elija through serial imitation and made use of 6 of the subjects (2 in each language), each of whom had previously responded to Elija's output in Experiment 1.

Experiments 1 & 2: First caregiver interactions with Elija

The first experiments investigated caregiver responses to Elija's 927 motor patterns. The caregivers were instructed to close their eyes and to imagine that they were interacting with a human infant. They were not given any information about the child's age, or shown a picture of an infant. They were asked to either respond or not respond 'naturally' to what they heard.

The caregivers prompted Elija to generate an utterance by pressing a key on the keyboard. Elija then executed a motor pattern, which generated a sound to which his caregiver might respond. Elija listened for 3 seconds after each of his productions and recorded any vocal response the caregiver chose to make. Elija

detected if the caregiver responded using a simple speech detection mechanism. This involved determining if the short-term power in any acoustic response exceeded background noise level. When a response was detected, the motor pattern responsible was retained and an association between the response and the motor pattern was created (Fig. 2). When a caregiver ignored a sound, the underlying motor pattern disappeared from Elija's motor pattern repertoire. Fig. 6 shows how this process forms associations between motor and auditory memories: immediately after executing a motor pattern, Elija captures any response from the caregiver in auditory memory, retains the motor pattern in motor memory and builds an association between the two.

We note that Elija did not change his motor patterns as a result of interaction with his caregivers (the same approach as taken by Miura et al. [28]). They were only optimized during the initial self-supervised learning stage. This study compared the behavior of different caregivers and it was therefore important that all caregivers heard the same sounds so that comparisons of their responses could be made.

Analysis of caregiver response criteria

In order to compare sounds that were accepted with those that were rejected during interaction with Eljija, we constructed two datasets. In the first, we included the utterances to which all six caregivers (E1, E2, F1, F2, G1, G2) responded. In the second dataset, we included utterances to which three or fewer caregivers responded. We preferred this selection process over examining caregivers' responses individually, since at least 3 caregivers had to agree for candidate sounds for inclusion in the rejection set, and all had to agree for inclusion in the acceptance set. This made the selection robust to noisy decisions made by the caregivers and lead to good exemplars in both categories.

We analyzed the motor patterns corresponding to the sounds in both datasets in terms of their individual Maeda and voice source vocal tract control parameters.

Each motor pattern was composed of 4 vectors, and the second and third played the main role in sound production (in fact in the CV and VC patterns, they played the entire role; only CVVs used the additional third target vector).

For each pattern we calculated the difference between the second target value and first target value. We also computed overall motor efforts for the utterance (see Supplementary Material Appendix S2).

The mean and standard error of these difference values were then calculated across motor patterns within each dataset. These results were then plotted.

We performed a corresponding analysis of Elija's output speech utterances in tams of total power, length, low-frequency power (< 4 kHz), high-frequency power (> 4 kHz), The filtering was carried out using a 2-pole Butterworth filter.

Experiment 3: Word learning mechanisms in Elija

After Elija had learned the associations between his productions and adult forms made in response, he could attempt to imitate novel utterances made by the caregiver (Fig. 3). He parsed them in terms of previously heard responses and since these sounds had associations with his motor patterns, this process provided him with candidates for the reproduction of words by serial matching of their component sounds.

To implement the recognition mechanism, Elija employed a template-based dynamic time warping (DTW) recognizer [26], running with an auditory gamma-tone filter bank front-end [5]. Such DTW recognizers typically operate by matching spectral representations of input speech with another set of such representations that correspond to the vocabulary of the recognizer. The latter are simply 'templates' or good examples of the sounds in its vocabulary. The template that gives the closest match is then taken as being the classification of the input sound. In the Elija model, the DTW recognizer used the caregiver's responses as its sound templates. However, since words could contain several basic speech sounds concatenated together, a segmentation mechanism was used to present them individually to the template-based recognizer. This required that the caregiver spoke with pauses between syllables. Segmentation into separate utterances was achieved by finding regions in which the short-term power of the signal exceeded the background noise level.

In practice, a two-pass recognition scheme was used to ensure real-time operation, as explained above. In the first pass, the recognizer operated by using 100 templates selected as the cluster centers of all responses. In the second pass, all the members of the best 5 clusters were used as templates. We note here that because Elija only matched caregiver speech with caregiver speech, there was no normalization problem for the classifier to solve.

During this experiment, Elija played out the motor patterns he had identified by the recognition process. Elija was given the ability to produce an intonation contour on each word resembling that of the caregiver, which made his attempts at word imitation sound more natural. To achieve this, the fundamental frequency contour for each separate speech sound was computed and approximated to a straight line using linear regression. The start and end frequencies were extracted and then mapped onto the range of the Maeda synthesizer voice source F0 parameter by assuming a linear scaling between the (-0.9, 0.9) parameter range and a frequency range of either 100 Hz-300 Hz or 150 – 400 Hz, for a male or female caregiver respectively. The duration of the speech sounds in the caregiver's speech was estimated and the values were limited to fall within the range of 250 ms – 600 ms. The F0 and duration parameter values were then used to set the fundamental frequency and duration parameters in the appropriate motor patterns. All interactions, including Elija's internal recognition process, were recorded to document the development of his pronunciation.

The word-learning task was run on a PC and a graphical user interface provided the caregiver with a word from a list, generated from words typically spoken by young children in the caregiver's language. The caregiver first pressed the GO

button and spoke the word. Elija then repeated it using his serial imitation mechanism. He could have up to 4 attempts at imitation, each of which could be selected in the user interface. The caregiver accepted or rejected Elija's responses by clicking on appropriate buttons. An important aspect of this infant-caregiver interaction was that they could engage in repetitive loops (Fig. 4). The word spoken by the caregiver could be repeated, which sometimes provoked a better response. This could continue until Elija performed an acceptable production, or the caregiver chose to give up and try another word.

References

1. Howard IS, Messum P (2011) Modeling the development of pronunciation in infant speech acquisition. Motor Control 15(1): 85-117.

2. Maeda S (1990) Compensatory articulation during speech: evidence from the analysis and synthesis of vocal tract shapes using an articulator model. In Hardcastle WJ, Marchal A, editors. Speech production and speech modeling. Boston: Kluwer Academic Publishers. pp. 131–149.

3. Maeda S (1979) An articulatory model of the tongue based on a statistical analysis. The Journal of the Acoustical Society of America, 65(S1): S22-S22

4. Saltzman E, Munhall K (1989) A dynamical approach to gestural patterning in speech production. Ecological Psychology 1(4): 333-382.

5. Slaney M (1993) An efficient implementation of the Patterson-Holdsworth auditory filter bank. Apple Computer, Perception Group, Tech. Report 35: 8.

6. D. P. W. Ellis (2009) Gammatone-like spectrograms, web resource. Available at http://www.ee.columbia.edu/~dpwe/resources/matlab/gammatonegram/

7. Messum PR (2007) The Role of Imitation in Learning to Pronounce. PhD Thesis, University of London. Available: https://sites.google.com/site/pmessum/downloads/.

8. Ellis D (2003) Dynamic Time Warp (DTW) in Matlab. Available at http://www.ee.columbia.edu/~dpwe/ resources/matlab/dtw/.

9. Lindblom BEF, Sundberg JEF (1971) Acoustical consequences of lip, tongue, jaw, and larynx movement. The Journal of the Acoustical Society of America, 50(4B): 1166-1179.

10. Fant G, Liljencrants J, Lin Q (1985) A four-parameter model of glottal flow.

STL-QPSR 4: 1-13.

11. Boë LJ, Heim JL, Honda K, Maeda S, Badin P (2007) The vocal tract of newborn humans and Neanderthals: Acoustic capabilities and consequences for the debate on the origin of language. A reply to Lieberman (2007a). Journal of Phonetics, 35(4), 564-581.

12. Markey KL (1994) The sensorimotor foundations of phonology: a computational model of early childhood articulatory and phonetic development. PhD Thesis, University of Colorado.

13. Flash T, Hogan N (1985) The coordination of arm movements: an experimentally confirmed mathematical model. The Journal of Neuroscience 5(7): 1688-1703.

14. Howard I, Messum P (2007) A Computational Model of Infant Speech Development. In XII International Conference "Speech and Computer" (SPECOM'2007) Moscow State Linguistics University. pp. 756-765.

15. Warlaumont AS (2013) Salience-based reinforcement of a spiking neural network leads to increased syllable production. In: Development and Learning and Epigenetic Robotics (ICDL), 2013 IEEE Third Joint International Conference. pp. 1-7.

16. Singh S, Lewis RL, Barto AG, Sorg J (2010) Intrinsically Motivated Reinforcement Learning: An Evolutionary Perspective. Autonomous Mental Development, IEEE Transactions 2: 70–82.

17. Moulin-Frier C, Oudeyer PY (2012) Curiosity-driven phonetic learning. In: Development and Learning and Epigenetic Robotics (ICDL), 2012 IEEE International Conference. pp.1-8.

18. Baranes A, Oudeyer PY (2013) Active learning of inverse models with intrinsically motivated goal exploration in robots. Robotics and Autonomous Systems 61: 49-73.

19. Rolf M, Steil JJ, Gienger M (2010) Goal babbling permits direct learning of inverse kinematics. Autonomous Mental Development, IEEE Transactions 2(3): 216-229.

20. Moulin-Frier C, Nguyen SM, Oudeyer PY (2013) Self-organization of early vocal development in infants and machines: the role of intrinsic motivation. Frontiers in Psychology 4:1006.

21. Warlaumont AS, Westermann G, Buder EH, Oller DK (2013) Prespeech motor learning in a neural network using reinforcement. Neural Networks

38: 64–75.

22. Yoshikawa Y, Asada M, Hosoda K, Koga J (2003) A constructivist approach to infants' vowel acquisition through mother–infant interaction. Connection Science 15: 245–258.

23. Stevens KN (1989) On the quantal nature of speech. Journal of Phonetics 17: 3-46.

24. Gunnilstam O (1974) The theory of local linearity. Journal of Phonetics 2: 91- 108.

25. Nabney I (2002) NETLAB: algorithms for pattern recognition. Springer.

26. Turetsky R, Ellis D (2003) Ground-truth transcriptions of real music from force-aligned midi syntheses. ISMIR 2003. pp. 135-141.

27. Sakoe H, Chiba S (1978) Dynamic programming algorithm optimization for spoken word recognition. Acoustics, Speech and Signal Processing, IEEE Transactions on 26(1): 43-49.

28. Miura K, Yoshikawa Y, Asada M (2012) Vowel Acquisition Based on an Auto-Mirroring Bias with a Less Imitative Caregiver. Advanced Robotics 26: 23–44.

Appendix S2: Analysis of caregiver responses

We investigated if differences in Elija's utterances and their corresponding motor patterns could explain why they were responded to (i.e. selected) or ignored (abandoned), using data from the six caregivers (E1, E2, F1, F2, G1, G2). We note that we only used 2 of the 4 English speakers for this analysis because this subset of responses was used later for the final word imitation experiments.

Methods

We first examined all Elija's utterances (927) in terms of the syllabic form of their motor patterns. We found there were 783 CVs, 111 VVs and 33 VCs. To make our results easier to interpret, we only analyzed characteristics of the utterances for the dominant CV subset of Elija's utterances. This avoided averaging results across different structural forms, which could potentially hide trends in the data.

Each CV motor pattern was composed of 4 sup-patterns, and the second and third characterized the sound production. For each pattern we calculated the difference between the third sup-pattern articulatory target values and second sup-pattern articulatory target values. We also computed the overall motor efforts for the utterance (see Elija Methods).

A corresponding analysis was performed on Elija's output speech for average power, average low-frequency power (< 4 kHz), average high-frequency power (> 4 kHz) and salience; the filters used for the low and high frequency analysis were both 2-pole Butterworth.

We compared characteristics of CV utterances that were responded to by caregivers with those that were ignored. To do so, we first constructed three datasets. In the first, we included the utterances to which all six caregivers (E1, E2, F1, F2, G1, G2) responded. In the second, we included utterances to which three or more caregivers did not respond. We used this selection process rather than examining caregivers' responses individually, since it gave datasets that strongly exhibited response/ignore tendencies. This made the selection process robust to noisy decisions made by the caregivers and ensured the inclusion of representative exemplars in both categories. The third dataset consisted of all CV utterances.

Results

The mean and standard error (standard deviation / square root of the number of samples) of the motor target difference values were calculated across motor patterns within each dataset. Statistical significance between the two conditions (responded/not responded) was determined using an unpaired two-sample t-test, since each of the two conditions contained a different numbers of data points (Matlab function ttest2); results were considered to be significant when $p < 0.05$. The results are plotted in Fig. S1A.

We performed the corresponding conditional analysis of Elija's output speech utterances in terms of their power, duration, salient and effort. These results are shown in Fig. S1B.

On the basis of the vocal tract articulatory target difference data (Fig. S1A) and the analyses of the utterance speech data, as well as effort (Fig. S1B), we make the following observations regarding preferences of caregivers to respond to Elija's utterances.

The following were highly significant (P < 0.001):
P2: Tongue dorsum position (+ve moving backwards). Caregivers prefer to respond when Elija's tongue is moving backwards.
P4: Tongue apex position (+ve when tip going forward and body going up). Caregivers prefer to respond when the tongue is going backwards
P5: Lip height (open lip is +ve). Caregivers prefer to respond when lips are opening
P6: Lip protrusion (+ve outwards). Caregivers prefer to respond when the lips are moving towards protrusion
P9: Fundamental frequency (higher frequency +ve). Caregivers prefer to respond when the intonation is falling
P10: Nasality (+ve open). Caregivers prefer to respond when there is decreasing nasality
Utterance power: Caregivers prefer to respond to utterances with higher overall power
Utterance low-frequency power: Caregivers prefer to respond to utterances with higher LF power
Temporal duration: Caregivers prefer to respond to utterances with shorter durations within the range that Elija generated. Utterances they responded to had a mean duration of 0.75 seconds whereas those they ignored had a mean duration of 0.85 seconds. Both durations are rather long for a syllable, so we speculate that the shorter durations were preferred because, other things being equal, they sounded more natural.
Effort: Caregivers prefer to respond to CVs, which involve more effort (voicing and articulatory movement)

The following was significant (P < 0.01):
P1: Jaw position (–ve closing). Caregivers prefer to respond when the jaw is opening.

The following were significant (P < 0.05):
P7: Larynx height (up +ve). Caregivers prefer to respond when the larynx is moving down
Utterance high-frequency power: Caregivers prefer to respond to utterances with more HF power

The following were not significant (P > 0.05):
P3: Tongue dorsum shape (+ve when higher). Had no significant effect.
P8: Glottal area (+ve louder voicing). Had no significant effect.
Salience: (+ve more salient). Had no significant effect. This result may seem surprising and we speculate that it is because the salience of the utterances was already optimized during the discovery process, and all utterances had therefore already achieved a high level of salience.

Conclusions

On the basis of the highly significant results ($p < 0.001$), it can be seen that for the CV utterances examined, caregivers were more likely to respond if Elija's mouth was opening and his lips were opening and protruding. This is unsurprising for a CV since a consonant generally involved some closure in the vocal tract, and a vowel is a more open configuration. Preference to reducing nasality is also understandable, since several consonants are nasalized, whereas fewer vowels are (only when followed by a nasal consonant in English and German). A preference for falling intonation was also observed, as was a preference for the tongue to move backwards during the utterance. Higher acoustic power, especially at lower frequency, was also preferred. Surprisingly, the salience measure was not a factor for preferential selection. A bias towards utterances that involved more effort on Elija's part was also apparent. This suggests a preference for dynamically changing utterances that involved articulator movement.

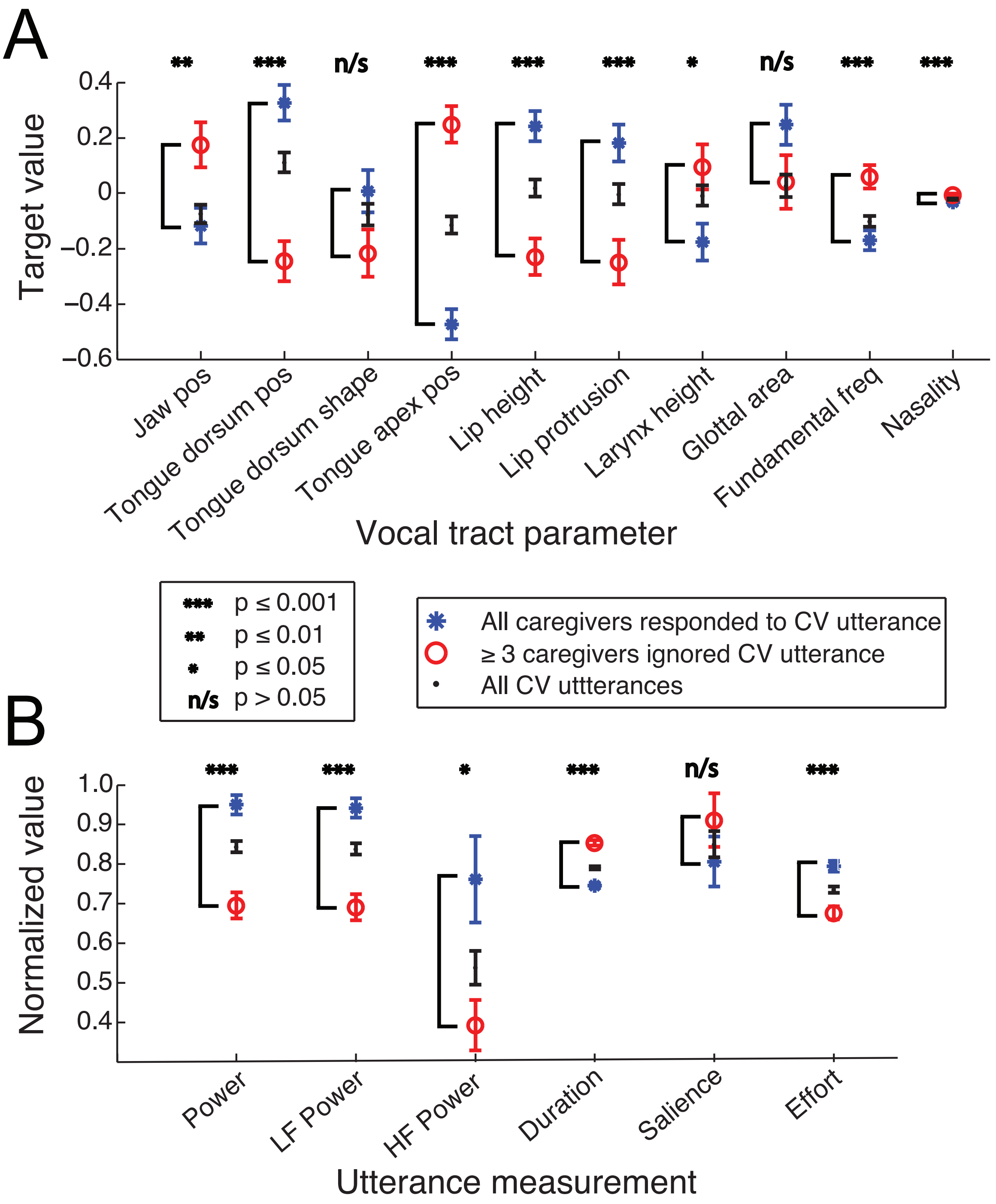

Figure S1. Analyses of Elija's CV utterances in terms of those responded to by all 6 caregivers and those ignore by at least 3 caregivers. Corresponding values for all CV utterances are also shown. **A** Plot of differences in the 3rd and 2nd motor pattern target parameters. **B** Plot of sound output characteristics as well as overall articulatory effort.

Supplementary material: Ian S. Howard & Piers Messum, PLOS ONE 2014
Learning to pronounce first words in three languages: an investigation of caregiver and infant behavior using a computational model of an infant

Appendix S3a: English Words (n=219)

hat
fin-ger
o-range
tick tock
bot-tle
brush
bis-cuit
uh-oh
ouch
bowl
meat
duck-ling
nose
duck
lion
hair
pigs
goat
house
this
mum-my
box
ba-nana
wat-er
hand
moon
tab-le
cat
look
app-le
egg
name
kit-ty
where
bee
lamp
cot
grand-pa
bath
fish
carr-ot
frog
out-side
boot
keys
pia-no
ma-ma
sock
pen-cils
sit
dad-dy
sheep
sun
chick
sky
slide
goose
shoe
train
torch
lips
man
mouse
crane
clean
cow
ice
thread
gi-raffe
baa baa
down
pear
tail
shin-y
poles
foot
bread
tea
yum-yum
what
fire-man
mon-key
cher-ries
cup
rug
leg
do
on-ion
two
pic-ture
moo
read
drink
moun-tain
boat
tv
light
mouth
spoon
belly
juice
slip-pers
play
bubb-le
spade
ham-mer
bye
go
pan
tow-el
eye
shirt
dish cloth
bear
plate
tooth
tree
buy
milk
work
chair
clock
ted-dy
pret-ty
pup-py
lem-ons
trac-tor
fork
grass
toy
ba-by
cook-ie
dol-ly
la-dy
sciss-ors
drum
no
top
lad-der
dog-gy
chest
screws
string
dir-ty
leaf
rat-tle
bye-bye
paint brush
owl
glass-es
car
book
bird
truck
cand-le
wheel
pen-guins
brace-let
ze-bra
cloud
cake
stuck
yawn
win-dow
toe
paint
blue
pen
glue
chick-en
drill
cam-el
dress
blocks
bun-ny
hel-lo
plane
worm
ear
big
flow-er
bus
wash
iron
bed
three
red
all gone
open
boy
grapes
dog
laugh
horse
knife
up
coat
yuk-ky
meow
yel-low
walk
gran-ny
arm
ber-ries
door
snake
tig-er
cry
eat
salt
broom
need-le
beak-er
bal-loon
comb
cheese
bead
tyres
green

Supplementary material: Ian S. Howard & Piers Messum, PLOS ONE 2014
Learning to pronounce first words in three languages: an investigation of caregiver and infant behavior using a computational model of an infant

Appendix S3b: French Words (n=219)

cha-peau
doigt
o-range
tic-tac
bou-teille
brosse
bis-cuit
oh-la
aie!
bol
viande
cane-ton
nez
ca-nard
lion
che-veux
porc
chevre
mai-son
ce-ci
ma-man
boite
ba-nane
eau
main
lune
table
chat
re-gardes!
pomme
oeuf
nom
mi-nou
ou
a-beille
lampe
ber-ceau
grand-pere
bain
pois-son
car-rotte
gre-nouille
de-hors
botte
clef
pia-no
ma-ma
chaus-sette
cra-yon
assieds-toi!
pa-pa
mou-ton
so-leil
pous-sin
ciel
glisses
oie
chaus-sure
train
torche
levres
homme
sou-ris
crane
nette
vache
glace
fil
gi-rafe
baa-baa
en bas
poire
queue
bril-lant
poles
pied
pain
the
mmm
quoi
pom-pier
singe
ce-rises
tasse
ta-pis
jambe
fais le
oi-gnon
deux
ta-bleau
meu
lis le
bois
mon-taigne
ba-teau
tele
lu-miere
bouche
cuil-lere
ventre
jus
pan-toufles
jeu
bulle
pelle
mar-teau
au (re)voir
vas
poele
ser-viette
oeil
che-mise
tor-chon
ours
plat
dent
arbre
ach(e)ter
lait
tra-vail
chaise
hor-lorge
our-son
jo-lie
chiot
ci-tron
trac-teur
four-chette
pe-louse
jou-et
be-be
ga-teau
pou-pee
dame
sci-seaux
tam-bour
non
en haut
e-chelle
poi-trine
vis
fi-celle
salle
feuille
ho-chet
bye-bye
pin-ceau
hi-bou
lu-nettes
voi-ture
livre
oi-seau
ca-mion
chan-delle
rouÈ
pin-guins
brace-let
zebre
nu-age
ga-teau
coin-ce
baille
fe-netre
or-teil
peinte
bleu
sty-lo
colle
pou-let
per-ceuse
cha-meau
robe
cubes
la-pin
bon-jour
a-vion
ver
o-reille
grand
fleur
bus
lave
fer
lit
trois
rouge
fini
ouvre
gar-con
rai-sins
chien
ri-gole
che-val
cou-teau
en haut
man-teau
beugh
miaou
jaune
marches
me-mere
bras
baies
porte
ser-pent
tigre
pleures
manges
sel
ba-lai
ai-guille
verre
ba-lon
peigne
fro-mage
perle
pneus
vert

Appendix S3c: German Words (n=237)

Möh-re	Qual-le	Blume	nein	lek-ker	Uhr
Sä-ge	Bus	Schärf	Stahl	Stift	Hut
Trak-tor	Lö-we	Stuhl	Luf-t	spiel	Blei-stift
Zahn	ach-tung	Sac-ke	Ja-cke	Flug-zeug	Nas-horn
Wal	e-ssen	Niko-laus	Bi-ene	Ast	Ball
Ze-bra	Rei-fen	Tisch	Schrau-be	Stie-fel	Gans
du	Kind	lau-fen	Hüh-ne	Fern-zeher	Bürs-te
Bock	Fla-sche	gut	Lam-pe	Saft	Pferd
Eli-ja	Ku-chen	Schmut-zig	Hand	In-sel	lachen
Wurm	Fuss	Ge-schenk	Maus	Brot	Fah-rrad
Mil-ch	Ko-ffer	durst	Mond	Wei-nen	Mantel
Ei-sen	Bär	Do-se	tun	Kra-bbeln	Alu
Au-to	Spiel	ja	Zan-ge	ich	Haar
pri-ma	Mund	tschüss	Ja-guar	So-nne	Kä-se
Han-dy	Bad	Ot-te	Au-ge	Knopf	Salz
Schnec-ke	Zwie-bel	Bein	Jan	Lol-li	oben
Ke-ks	Gras	er	Jo-Jo	Korb	set-zen
gross	Wol-ke	Baum	Kis-te	Buch	Drei-rad
run-ter	Kä-fer	Brust	Nest	Arm	Jun-ge
Ho-se	Hemd	Fenster	Lö-ffel	Fro-sch	Os-ter
Ti-ger	Schirm	wo	Ei	für	Amsel
Tee	Men-sch	Trommel	Hi-mmel	Kleid	Hund
Band	Vase	Schlü-ssel	Zug	Schwein	Flö-te
O-range	Rol-ler	Rech-ner	Ka-nne	al-le	Was-ser
Hu-tte	Kuh	Ge-tränk	Ki-ssen	Mu-tti	mü-de
Vo-gel	Un-ter	das	Streich	Stoff	Erb-se
Del-fin	Tas-se	Zelt	Trau-ben	Bech-er	O-ber
Ker-ze	Kat-ze	was-chen	Vul-kan	Öl	See
drau-ssen	Fin--ger	A-ffe	Ohr	fahr-en	Ted-dy
Blatt	Teller	Stern	Na-se	Boot	Trich-ter
hun-ger	Be-sen	Uh-le	Bla-sen	Clown	Lipp-en
Ma-ske	Pfa-nne	Ra-be	Wür-fel	Re-gen	spiel-en
Bett	Gu-mmi	Hammer	Pin-guin	Bal-lon	Kir-sch
Draht	Na-gel	ka-ffee	Kran	Piers	bit-te
Hase	Mu-tter	toll	Erd-beer	Sie	Schei-be
Pa-pi	Fisch	Wal-ross	Tür	Spi-nne	ha-llo
Schuh	Bahn	Jo-ghurt	Bir-ne	rut-chen	Me-sser
Boh-rer	Ap-fel	Kamm	Ka-mel	Pfe-ffer	
I-gel	Yak	Ente	Geh	Ga-bel	
Sche-re	Kle-ber	Schü-ssel	schlecht	Pu-ppe	

Appendix S4: Word imitation analysis

Comparisons for all subjects between archiphoneme representations of caregiver target words and Elija's imitations

Fig. 13 in the main paper shows comparisons between the speech sounds in the caregivers' word productions and the speech sounds in Elija's imitations. The latter were already labeled previously since they were established during the first interaction experiment. The speech sounds were analyzed in terms of first vowels V_1 and consonants C_1. The results are presented individually for each of the 6 caregivers. This data is analyzed further here.

The overall behavior of each caregiver is shown in Fig. S2. This indicates how many time the caregiver and Elija's imitation had the same archiphoneme labeling. It can be seen that the archiphonemes representing Elija's imitations were closer to the caregiver's word productions for vowels (**A**) than consonants (**B**, apart from English speaker E1), particularly for the German speakers G1 and G2.

The 95% confidence intervals on Fig. S2 are quite large due to the relatively small number of data counts in each condition. We note that the assumption of normal distribution was valid as there were $np \geq 10$ samples in each condition, with the exception of E2 (only 8 values). However we did not pursue this issue further and the error bar is displayed only to give a rough indication of the general variability of the data.

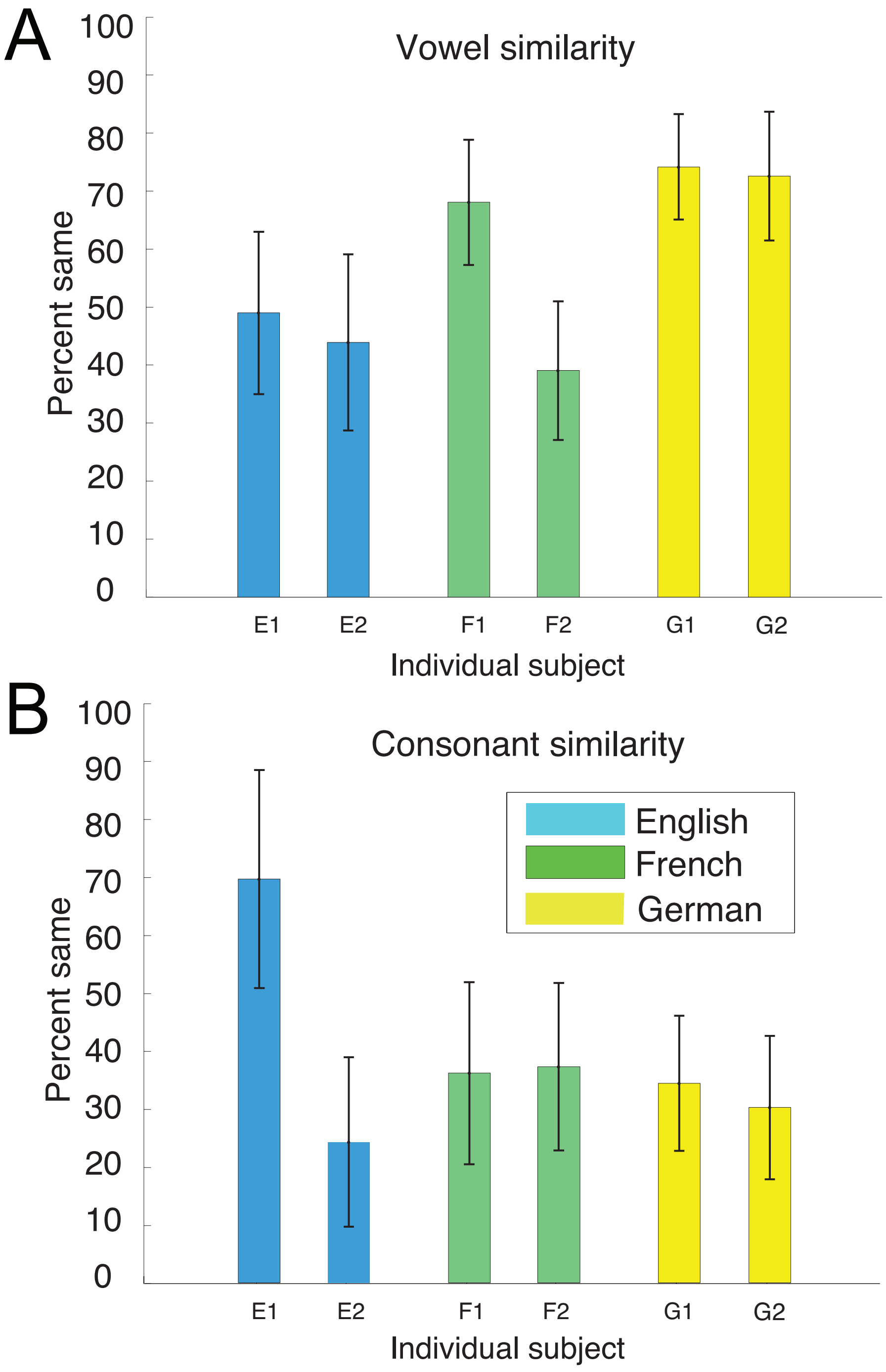

Figure S2. Comparisons for all subjects between archiphoneme representations of caregiver target words and Elija's imitations. Comparisons between caregiver's words and Elija's imitations by **A** vowel and **B** consonant. The error bars show 95% confidence intervals

Appendix S5: Online Data repository

All the experimental data arising from Elija and his interactions with caregivers is available online at:

https://github.com/HowardLab/Elija-PlosOne-2014.git

This repository is composed of the following directories:

Elija927DiscoveredSounds: This contains all 927 of Elija's discovered sounds as WAV files

ElijaMPs: This contains all 927 motor patterns for the discovered sounds saved as doubles in .txt files

A motor pattern is a sequence of articulatory parameters to control the Maeda synthesizer. Each motor pattern consists of a file containing 138 floating-point values, although a few values are unused. It contains 4 sub-patterns, each consisting of 10 articulation target positions and their associated movement parameters; namely their starting times and durations. That is, there are 4 sets of 10 x target values, 10 x starting times, 10 x durations (we note that the first two sub-patterns were often identical, so the motor pattern generally consisted of three different sub-patterns). In addition each sub-pattern has a critical damping beta scaling value.

The ten articulatory parameters are ordered as follows:

P1 Jaw position
P2 Tongue dorsum position,
P3 Tongue dorsum shape,
P4 Tongue apex position,
P5 Lip height (aperture),
P6 Lip protrusion,
P7 Larynx height.
P8 Glottal area,
P9 Fundamental frequency
P10 Nasality

After the motor parameters in a given .txt file are read into the linear array vtParams, using Matlab notation, they can be accessed as follows:

The 4 sets of 10 x target values are given by:
Target vector 1: vtParams(1:10);
Target vector 2: vtParams(11:20);

Target vector 3: vtParams(21:30);
Target vector 4: vtParams(31:40);

The 4 sets of 10 x start times are given by:
Start times vector 1: vtParams(51:60);
Start times vector 2: vtParams(61:70);
Start times vector 3: vtParams(71:80);
Start times vector 4: vtParams(81:90);

The 4 sets of 10 x durations are given by:
Duration vector 1: vtParams(91:100);
Duration vector 2: vtParams(101:110);
Duration vector 3: vtParams(111:120);
Duration vector 4: vtParams(121:130);

The 4 sets of single beta scale components are given by:
Beta scale vector 1: vtParams(131);
Beta scale vector 2: vtParams(132);
Beta scale vector 3: vtParams(133);
Beta scale vector 4: vtParams(134);

ElijaSoundInteractions: This contains data from Elija's initial interaction with a caregiver. It consists of Elija's productions and their corresponding responses (if any) for each caregiver. Response data is identified by the subject description used in the main manuscript: E1, E2, E3, E4-1, E4-2, E4-3, E4-4. F1, F2, G1. G2.

WordImitationExperiments: This contains WAV data from the word imitation experiments. This consists of the sound files of caregivers' word production, how Elija recognized them in terms of reformulations, and also Elija's word imitation productions. Word learning data is identified by the subject description used in the main manuscript: E1, E2, E3, F1, F2, G1, G2 The English, French and German word lists are also included in this directory.

PowerPoint presentation: ElijaOutputDemo2014.pptx is a PowerPoint presentation of some of the material.

vtsynth: This contains a Windows VC++ project implementation of the vocal tract synthesizer that can be called from Matlab 7.1 running on Windows XP

Elija_Matab : This contains Matlab files illustrating:

Supplementary material: Ian S. Howard & Piers Messum, PLOS ONE 2014
Learning to pronounce first words in three languages: an investigation of caregiver and infant behavior using a computational model of an infant,

Vowel discovery process - Main_RunActiveLearn_VOWEL_DEMO.m
Running this script will lead to the discovery of vocalic sounds.

Fricative discovery process - Main_RunActiveLearn_FRIC_DEMO.m
Running this script will lead to the discovery of fricative sounds.

Closure discovery process - Main_RunActiveLearn_CLOSURE_DEMO.m
Running this script will lead to the discovery of vocal tract closures.

The reformulative interaction experiment - Main_RunInteractReformulations.m
Running this script will use the set of 927 discovered utterances used in the 1st interaction excrement and the caregiver has the opportunity to either respond or ignore them.

In addition, all the necessary Matlab functions called by these scripts are also included.

Please note that no support from the authors is available for any of the online context. It is provided to assist readers to understand the Elija model and to demonstrate its underlying operation, and to provide examples of the interactions of Elija and the caregivers described in the main PLOSONE article.
