# Supplementary figures and images for: Learning to Pronounce First Words in Three Languages: An Investigation of Caregiver and Infant Behavior Using a Computational Model of an Infant
Source: PLoS One. 2014 Oct 21;9(10):e110334. doi: 10.1371/journal.pone.0110334 (PMC4204867; doi:10.1371/journal.pone.0110334)

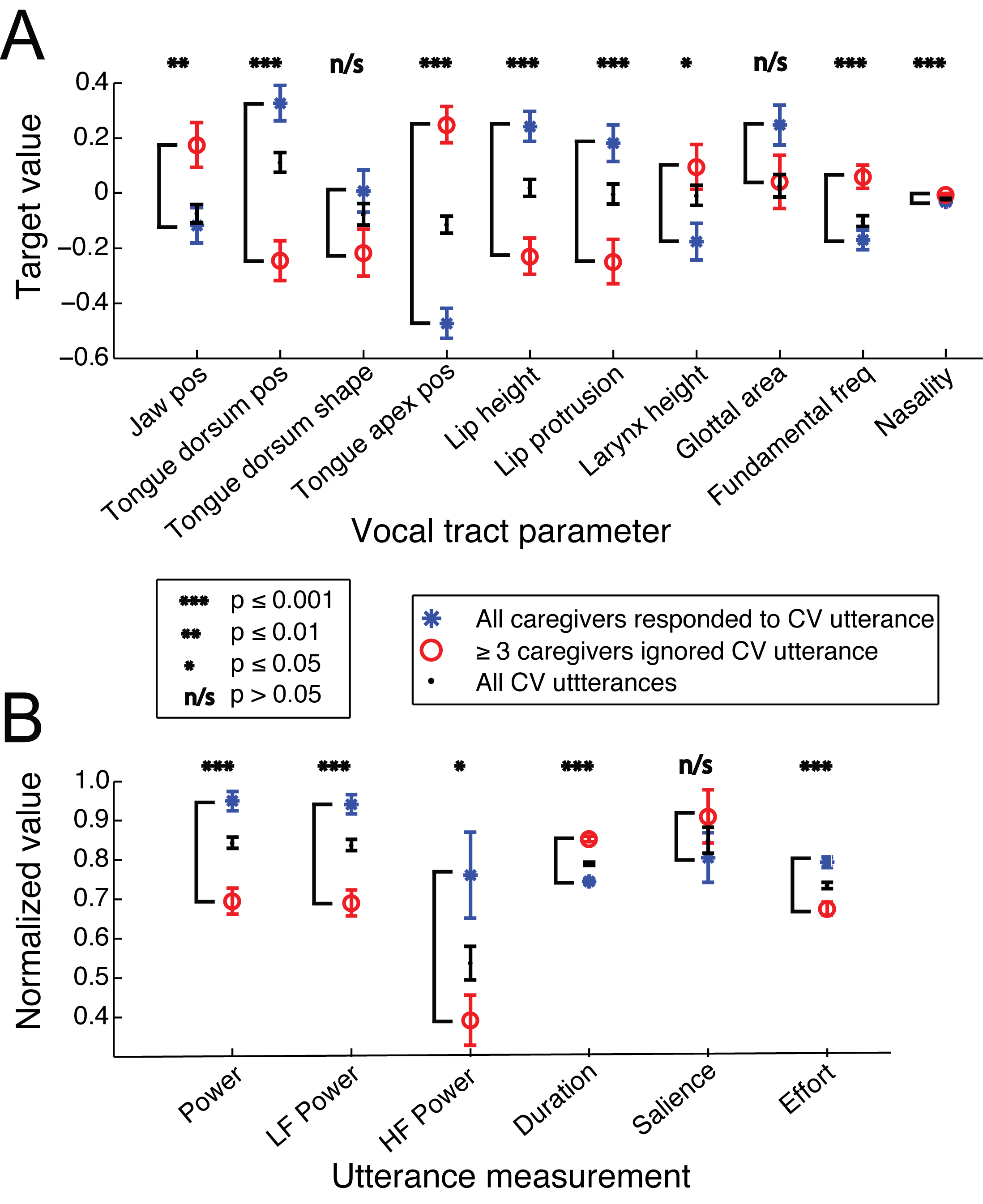

Supplement: Figure S1 — (TIF) [file pone.0110334.s002.tif]

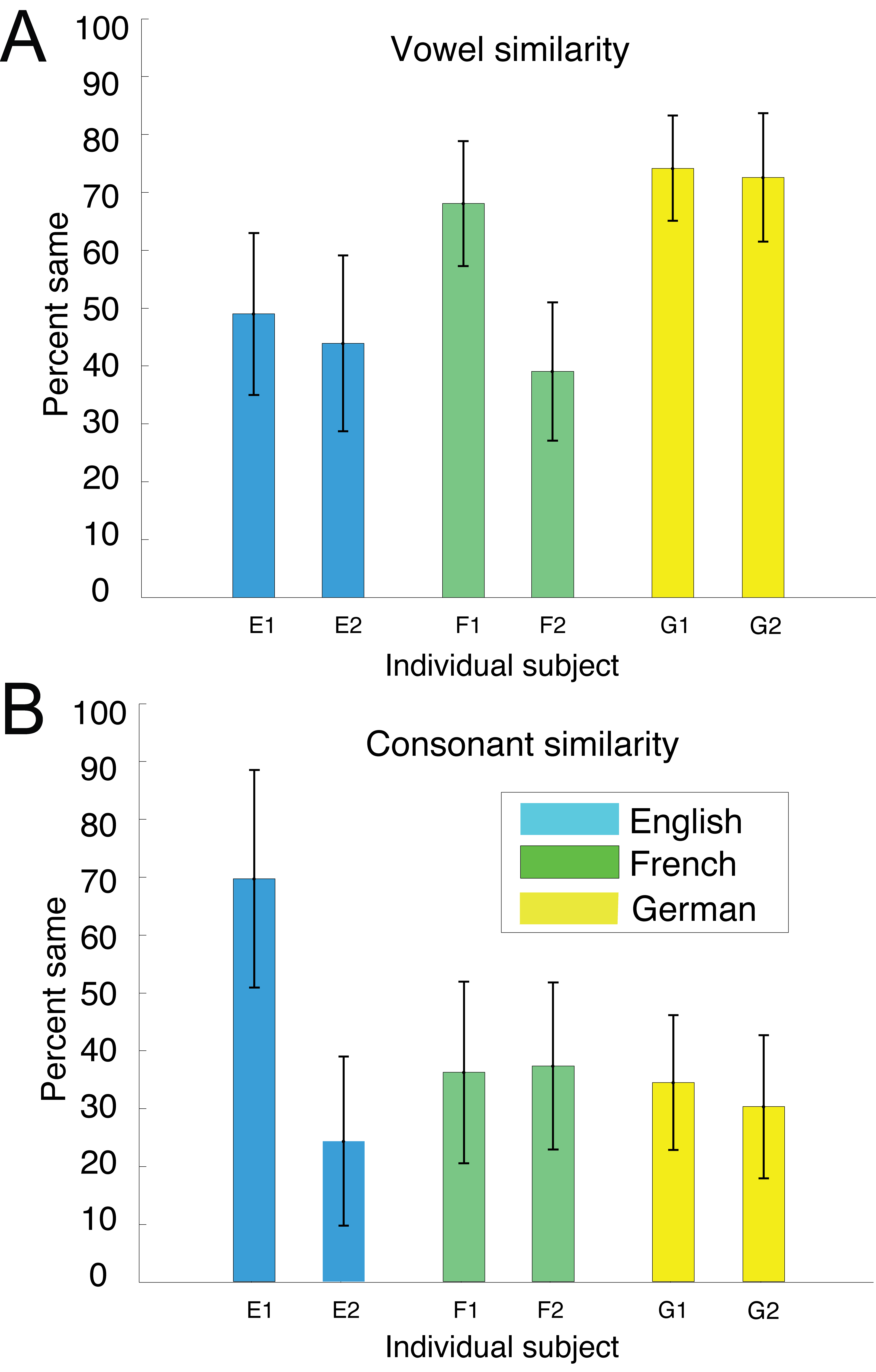

Supplement: Figure S2 — (TIF) [file pone.0110334.s003.tif]
